# Heterogeneous effects of hospital competition on inpatient expenses: an empirical analysis of diseases grouping basing on conditions’ complexity and urgency

**DOI:** 10.1186/s12913-021-07331-1

**Published:** 2021-12-10

**Authors:** Liyong Lu, Xiaojun Lin, Jay Pan

**Affiliations:** 1 HEOA Group, West China School of Public Health and West China Fourth Hospital, Sichuan University, No. 17, Section 3, Ren Min Nan Road, Chengdu, 610041 Sichuan China; 2 Institute for Healthy Cities and West China Research Center for Rural Health Development, Sichuan University, Chengdu, China

**Keywords:** Hospital competition, K-means clustering, Predicted patient flow approach, Inpatient expense, China

## Abstract

**Background:**

Multiple pro-competition policies were implemented during the new round of healthcare reform in China. Differences in conditions’ complexity and urgency across diseases associating with various degrees of information asymmetry and choice autonomy in the process of care provision, would lead to heterogeneous effects of competition on healthcare expenses. However, there are limited studies to explore it. This study aims to examine the heterogeneous effects of hospital competition on inpatient expenses basing on disease grouping according to conditions’ complexity and urgency.

**Methods:**

Collecting information from discharge data of inpatients and hospital administrative data of Sichuan province in China, we selected representative diseases. K-means clustering was used to group the selected diseases and Herfindahl-Hirschman Index (HHI) was calculated based on the predicted patient flow to measure the hospital competition. The log-linear multivariate regression model was used to examine the heterogeneous effects of hospital competition on inpatient expenses.

**Results:**

We selected 19 representative diseases with significant burdens (more than 1.1 million hospitalizations). The selected diseases were divided into three groups, including diseases with highly complex conditions, diseases with urgent conditions, and diseases with less complex and less urgent conditions. For diseases with highly complex conditions and diseases with urgent conditions, the estimated coefficients of HHI are mixed in the direction and statistical significance in the identical regression model at the 5% level. For diseases with less complex and less urgent conditions, the coefficients of HHI are all positive, and almost all of them significant at the 5% level.

**Conclusions:**

We found heterogeneous effects of hospital competition on inpatient expenses across disease groups: hospital competition does not play an ideal role in reducing inpatient expenses for diseases with highly complex conditions and diseases with urgent conditions, but it has a significant effect in reducing inpatient expenses of diseases with less complex and less urgent conditions. Our study offers implications that the differences in condition’s complexity and urgency among diseases would lead to different impacts of hospital competition, which would be given full consideration when designing the pro-competition policy in the healthcare delivery system to achieve the desired goal.

**Supplementary Information:**

The online version contains supplementary material available at 10.1186/s12913-021-07331-1.

## Introduction

In recent decades, competition was introduced in the healthcare market across countries, such as Germany, France, and the United Kingdom, with the objective of improving the efficiency of health care services [1–7]. The ongoing reform in China aims at promoting access to appropriate health care and controlling cost inflation. Multiple pro-competition policies including enlarging operational autonomy of public hospitals, and encouraging private hospital development [8–10], were also implemented during the reform to improve the efficiency of the healthcare delivery system in China [11].

With the implementation of pro-competition policies, the competition impact on healthcare expense has become a hot topic that received considerable attention in recent years [12–15]. The dispute about the effectiveness of competition in the healthcare market mainly stems from the particularities of the healthcare market [16]. Classical economics clearly predicts market competition would inspire the industry to reduce the value-adjusted price under specific prerequisites. Among them, consumers with enough information about the products or services can freely choose the providers as one of the main premises [17]. However, highly asymmetric information between physicians and patients, and patient’s limited choice autonomy in service selection exists in the healthcare market [16, 18], complicating the effectiveness of competition [19, 20].

The particularities of highly asymmetric information and patient’s limited choice autonomy in the healthcare market would reflect in the process of care provision associating with disease conditions. Patients with highly complex disease conditions, such as multiple complications, would have difficulties in obtaining or understanding the disease and treatment-related knowledge, leading these patients to face greater information asymmetry than the patients with common diseases. Patients who suffer from the disease with urgent conditions, such as acute myocardial infarction, need to be admitted to the hospital for treatment within a very short time, generally selecting the nearest hospital according to treatment capacity, leaving them or their companions limited opportunity to choose hospitals [21]. The prerequisite for market competition to play a role is missing in the above two kinds of diseases, leading to the uncertain impacts of competition for these diseases in the healthcare market. For the diseases of which conditions are generally less complex and less urgent, the information asymmetry and patient’s limited choice autonomy in the process of care provision would not be obvious. The services for these diseases would like the products in the “textbook market”, and competition effectiveness would be predicted by the classical economic theory.

Differences in conditions’ complexity and urgency across diseases associating with various degrees of information asymmetry and choice autonomy in the process of care provision, would lead to heterogeneous effects of competition on healthcare expenses. However, there are limited studies to explore it. Most of the previous studies have not considered the influences of differences in healthcare characteristics [12, 13, 15, 22, 23]. To our knowledge, C Deng and J Pan [19] is the only study considering the influences of differences in healthcare characteristics on the effects of hospital competition on healthcare expenses. Prostatectomies (elective surgery, representing treatments of non-acute common diseases) and appendectomies (emergency surgery, representing treatments of acute common diseases) are selected as the representative surgeries, and they found that greater hospital competition was significantly associated with lower total hospital charges for prostatectomies, while the opposite was true for appendectomies.

To bridge the gap in the literature, our study aims to explore the heterogeneous effects of hospital competition on inpatient expenses basing on disease grouping according to condition’s complexity and urgency. The significance of considering the heterogeneous effects of hospital competition among different diseases groups are as follows: (1) The findings of this study can provide empirical evidence for the influence of disease conditions on the effects of competition, indirectly examining the effects of hospital competition in various degrees of particularities in the healthcare market. (2) To achieve the pro-competition policies’ optimal impact on healthcare systems while minimizing the potential adverse outcomes, the heterogeneous effects of hospital competition among different diseases groups are needed to explore to find out what kinds of disease hospital competition could not play an ideal role in reducing inpatient expenses, providing important policy implications.

## Methods

### Study area

This study is based on Sichuan province located in southwest China between 26.40°N and 33.68°N latitude, and 98.31°E and 107.99°E longitude [24]. The province consists of 183 counties, and it is China’s fifth-largest province (486,052 km2) in land size, with 83.41 million residents in 2018.

There are great variations in topography, economy, and population distribution within the Sichuan province [25, 26]. In 2018, the maximum population in a county (Wuhou county with 1.87 million residents) was reported more than 65 times of the minimum one (Derong county with 28 thousand residents), while the most developed county (Longquanyi county with GDP about 22,000 dollars per capita) was reported over 14 times compared with the lowest one (Shiqu county with GDP about 1500 dollars per capita). The differences in the distribution of topography, economy, and population among regions would lead to large diversities in the local healthcare development as well as the healthcare market. In 2018, Wuhou county with the most hospitals (109) had more than 20 health technicians per 1000 population, while Jinkouhe county had only 1 hospital with 5.09 health technicians per 1000 population. The uneven distribution of healthcare facilities and medical resources has brought about great variations in the degrees of hospital competition among different regions, thus providing us an ideal opportunity for testing our hypothesis^1^ [16, 25].

### Data source

We collected the patient-level information from the inpatient discharge dataset during the period of the fourth quarter of 2018 (from September to December), and hospital-level information from the hospital administrative data in 2018. Both data were managed by and obtained from Sichuan Provincial Health Statistics Support System Database. The inpatient discharge dataset contains patient-level information, including as follows [16, 27]: (1) The patient’s basic information includes their age, gender, and health insurance program (Urban Employment Basic Medical Insurance, Urban Residents Basic Medical Insurance, New Cooperative Medical Scheme, full self-expenses, and others). (2) The admission information includes the admission source (transferred from the outpatient department within the hospital, from the emergency department within the hospital, from other hospitals, and others), the urgency at admission (critical urgent, urgent, and general), International Classification of Diseases, 10th Revision (ICD-10) codes and the names of primary and secondary diagnoses. (3) The discharge information includes the discharge method (following the doctor’s advice to leave the hospital, following doctor’s advice to transfer to other hospitals, not following the doctor’s advice to leave the hospital, and others), the discharge status (cured, improved, unhealed, death, and others), and the inpatient expenses (total expenses and sub-group expenses). (4) The basic information of the hospitals that patients are admitted to (e.g., hospital identifier, hospital level). The hospital administrative data reported annually by hospitals at the end of the year contains detailed hospital-level characteristics, such as hospital identifier, administrative division code, geographic location, number of beds, staffing level, whether general, whether for-profit, and whether public hospital [27]. In addition to these two datasets, we derive the region-level demographic, socioeconomic, and health resource data from the Sichuan Health Statistical Yearbook and Sichuan Statistical Yearbook, including the number of health technicians per 1000 population, the number of residences, and the GDP per capita. The inpatient discharge dataset was linked to the hospital administrative data by the hospital identifiers and further linked to the demographic, socioeconomic, and health resource data by the administrative division codes of hospitals [27]. The latitude and longitude of the hospital and patient locations were geocoded from the Gaode map based on the hospital name and address, as well as the patient’s address, which was utilized to obtain the distance metrics among hospitals, and between hospitals and patients [26].

### Disease selecting

In this study, we focused on diseases with significant burdens. We used inpatient volume and medical expenses to reflect the disease’s burden [26]. According to the regulations in the authority file [28], the first three digits of the ICD-10 code can be used as a statistical classification, so we used 3 digits ICD-10 code to identify the observations in this study. Referring to previous study [26], the detailed process of disease selecting are as follows: (1) the diseases were arranged in descending order according to the inpatient volume, and the top 20 diseases with the largest inpatient volume were selected; (2) the diseases were arranged in descending order according to the total expenses of all inpatients, and the top 20 diseases were selected. This study also considers the characteristics of the diseases to ensure that the selected diseases could cover acute and chronic diseases, severe and mild diseases, as well as various departments (including orthopedics, gastroenterology, neurosurgery, endocrinology, etc.). Ultimately, 19 diseases were selected in this study, including bronchial and lung cancer (C34), non-insulin-dependent diabetes (E11), schizophrenia (F20), essential hypertension (I10), chronic ischemic heart disease (I25), cerebral infarction (I63), hemorrhoid (I84), pneumonia (J12-J18), acute bronchitis (J20), chronic obstructive pulmonary disease (J44), gastritis and duodenitis (K29), cholelithiasis (K80), spine joint stiffness (M47), intervertebral disc disorders (M50-M51), obstructive and reflux uropathy (N13), chronic kidney disease (N18), intracranial injury (S06), femoral fracture (S72), fractures of the lower leg (including the ankle) (S82),^2^ involving more than 1.1 million hospitalizations (about 35% of total hospitalizations) in our empirical analysis.

We cleaned up the data of 19 selected diseases before analysis. Table 1 shows the details of the data cleaning process.

**Table 1:** Selecting disease and data cleaning

Diseases	ICD-10 code	Criterion			
		Including all inpatients of this disease admitted to hospitals in Sichuan province in the fourth quarter of 2018	Excluding those individuals whose address missing	Excluding those individuals whose medical costs missing	Final data: number of inpatients
Bronchial and lung cancer	C34	20,559	47	1	20,511
Non-insulin dependent diabetes	E11	42,923	141	12	42,770
Schizophrenia	F20	47,230	193	15	47,022
Essential hypertension	I10	36,770	192	2	36,576
Chronic ischemic heart disease	I25	76,593	391	6	76,196
Cerebral infarction	I63	79,834	420	1	79,413
Hemorrhoid	I84	35,435	102	1	35,332
Pneumonia	J12-J18	180,191	551	21	179,619
Acute bronchitis	J20	62,861	392	11	62,458
Chronic obstructive pulmonary disease	J44	152,319	722	30	151,567
Gastritis and duodenitis	K29	58,593	433	11	58,149
Cholelithiasis	K80	59,288	143	6	59,139
Spine joint stiffness	M47	59,490	434	28	59,028
Intervertebral disc disorders	M50-M51	10,0511	746	1	99,764
Obstructive and reflux uropathy	N13	36,524	177	3	36,344
Chronic kidney disease	N18	24,081	74	0	24,007
Intracranial injury	S06	23,699	68	3	23,628
Femoral fracture	S72	17,327	79	1	17,247
Fractures of the lower leg (including the ankle)	S82	22,175	121	3	22,051

### Competition measurement

We calculated Herfindahl-Hirschman Index (HHI) for each disease to measure hospital competition. To address the potential endogeneity of conventional estimation of HHI, such as fixed radius and geopolitical boundaries approach, we employed the predicted patient flow approach to define the hospital market and to calculate HHI [32–34].

The predicted patient flow approach was proposed by DP Kessler and MB Mcclellan [35] and defines the potential market capturing the potentially competitive hospitals, rather than defining geographic markets arbitrarily [32–35]. This method calculates the expected patient shares based on exogenous determinants of patient flows, such as distance between patients and hospitals, rather than potentially endogenous indicators.

Specifically, referring to previous studies [32–35], the process to measure the hospital competition by predicted patient flow method is as follows:

First, we constructed the patient’s hospital choice model,^3^ and assume that patients choose the hospital that maximizes their utility. The choice model in this study mainly refers to [32, 35], and it is as follow^4^:

1 $${U}_{ij}=\sum \limits_{k=1}^3\left\{\begin{array}{l}{\beta}_1^k\left({d}_{ij}-{d}_{ij^{+}}^k\right)\times {z}_j^k+{\beta}_2^k\left({d}_{ij}-{d}_{ij^{+}}^k\right)\times \left(1-{z}_j^k\right)\\ {}+{\beta}_3^k\left({d}_{ij}-{d}_{ij^{-}}^k\right)\times {z}_j^k+{\beta}_4^k\left({d}_{ij}-{d}_{ij^{-}}^k\right)\times \left(1-{z}_j^k\right)\\ {}+{\beta}_5^k\left({female}_i\times {z}_j^k\right)\\ {}+{\beta}_6^k\left({age}_i\times {z}_j^k\right)\\ {}+{\beta}_7^k\left({highseverity}_i\times {z}_j^k\right)\\ {}+{\beta}_8^k\left(\mathrm{s}{erious}_i\times {z}_j^k\right)\\ {}+{\beta}_9^k\left({emergency}_i\times {z}_j^k\right)\\ {}+{\beta}_{10}^k\left({CCI}_i\times {z}_j^k\right)\end{array}\right\}+{e}_{ij}$$

Where *U*_*ij*_ denotes inpatient *i*’s indirect expected utility from choosing hospital *j*, as the sum of a function of the relative distances and hospital characteristics *Z*_*j*_^*1*^, *Z*_*j*_^*2*^, *Z*_*j*_^*3*^; a function of inpatient *i*’s demographic characteristics *female*_*i*_, *age*_*i*_, *lowseverity*_*i*_, *emergency*_*i*_ and *CCI*_*i*_ and hospital characteristics *Z*_*j*_^*1*^, *Z*_*j*_^*2*^, *Z*_*j*_^*3*^; and random error *e*_*ij*_. Among them, *Z*_*j*_^*1*^, *Z*_*j*_^*2*^, *Z*_*j*_^*3*^ represents whether hospital *j* is a public hospital, whether hospital *j* is a tertiary hospital, whether hospital *j* is a big hospital (defined as the actual number of hospital beds over the median bed for a specific disease), respectively; *female*_*i*_ and *age*_*i*_ denote the gender and age of inpatient *i*, respectively. *highseverity*_*i*_ is binary indicator of whether inpatient *i* has more than three diagnosis codes in their secondary diagnoses (then low severity as the reference group). *serious*_*i*_ is binary indicator of the inpatient *i*’s urgency at admission is critical urgent or urgent (general as the reference group). *emergency*_*i*_ indicates whether inpatient *i* is admitted through the emergency department (admission not from the emergency department as the reference group). *CCI*_*i*_ denotes Charlson Comorbidity Index (CCI) to reflect the complications of inpatients. ${d}_{ij}-{d}_{ij^{+}}^k$ is the distance from inpatient *i*’s residence to hospital *j* minus the distance from *i*’s residence to the nearest hospital with the same characteristics *Z*_*j*_^*1*^, *Z*_*j*_^*2*^, *Z*_*j*_^*3*^. ${d}_{ij}-{d}_{ij^{-}}^k$ is the distance from *i*’s residence to hospital *j* minus the distance from *i*’s residence to the nearest hospital with different characteristics in terms of *Z*_*j*_^*1*^, *Z*_*j*_^*2*^, *Z*_*j*_^*3*^ [32, 35] . According to the previous study, patient’s choice sets ***J*** was restricted to their chosen hospital and all hospitals within 100 km [32, 33, 35] (we also used different lengths to define the inpatients’ choice set ***J*** in the robust check).

Second, we predicted the probability of inpatient *i* admitted to hospital *j* in their choice set ***J*** based on the above hospital choice model.

2 $${\hat{P}}_{ij}=\frac{\exp \left({\hat{u}}_{ij}\right)}{\sum \kern0.5em \exp \left({\hat{u}}_{ij}\right)}$$

where ${\hat{u}}_{ij}$ is the utility of inpatient *i* admitting to hospital *j*.

Third, the HHI for inpatient *i* was calculated.

3 $${HHI}_i=\sum \limits_{j\in J}{\left({\hat{P}}_{ij}\right)}^2$$

Fourth, HHI for hospital *j* was calculated.

4 $${HHI}_i=\frac{1}{N_j}\sum \limits_{i\in I}\left({\hat{P}}_{ij}\ast {HHI}_i\right)$$

where *I* refers to these inpatients who might potentially choose hospital *j*, ${N}_j=\sum \limits_{i\in I}{\hat{P}}_{ij}$ indicates the expected volume of hospital *j*.

### Statistical analysis

#### Diseases grouping

Using K-means clustering, we classified the selected diseases. K-means clustering, a common unsupervised algorithm in machine learning, divides the data into different groups according to their characteristics [36]. We used the Hopkins statistic value to test the cluster forming tendency of the data [37, 38].^5^ The optimal number of clusters (K) was determined based on the sum of the squared error (SSE, also called the elbow method)^6^ [40]. Cluster analysis was performed on scaled and centered values. Cluster labels were assigned based on the characteristics of individual cluster mean values of the indicators [41].

According to the aims of this study, the clustering indicators were selected from the point of complexity and urgency of the disease conditions. This study used the disease-specific inpatient emergency admission rate to measure the urgency of the disease conditions. We calculated the disease-specific inpatient average Charlson Comorbidity Index (CCI) to reflect the complexity of the disease conditions. A detailed explanation of why we use disease-specific inpatient emergency admission rate and CCI as the clustering indicators are shown in the appendix text (Text 3) in the supplementary file.

#### Regression analysis

First, the univariate analysis was performed. The continuous variables were described by the means (M) and standard deviation (SDs), while the categorical variables by frequency and percentage.

Second, the effects of hospital competition on the inpatient expenses were analyzed by the log-linear multivariate regression model. The model is set as follows:

5 $$\log \left({Expense}_{idhm}\right)={\beta}_0+{\beta}_1{HHI}_{hm}+{P}_{idhm}\gamma+{A}_{idhm}\phi+{T}_{idhm}\varphi+{H}_{hm}\theta+{C}\rho+{\varepsilon}_{idhm}$$

where *m* denotes the market, *h* the hospital, *d* the disease, and *i* the inpatient. *Expense* is the explained variable, which denotes the inpatient expenses. The variable *HHI* is the key independent variable, which indicates the competition intensity (or concentration)*.*

*P* is a vector of patient’s basic characteristics, including gender, age, and their health insurance program. *A* is a vector of the patient’s admission characteristics, including the admission source, the urgency of admission. *T* is a vector of the patient’s treatment information, including the CCI. *H* is a vector of variables related to the hospital’s characteristics, including the hospital level, ownership, whether general, whether for-profit, and the number of beds. *C* is the vector of county characteristics, including the number of health technicians per 1000 population, the number of residences, and the GDP per capita.ε is the error term.

We use robust standard errors to correct heteroskedasticity. Due to the positively skewed distribution of inpatient expenses, the number of beds, the number of health technicians per 1000 population, the number of residences, and the GDP per capita, we applied their logarithmic transformations in the regressions.

*β*_*1*_ is the coefficient of interest. A positive value means that hospital competition would reduce the inpatient expenses (or hospital concentration would increase the inpatient expenses). All analyses were performed using STATA 15.0. *P* < 0.05 was used to determine statistical significance.

## Results

### Descriptive statistics

Table 2 displays the means and standard deviation of continuous variables, and the frequency and percentage of categorical variables.^7^

**Table 2:** Descriptive statistics for variables

Diseases Variables	C34	E11	F20	I10	I25	I63		I84	J12–18	J20
***Inpatient costs***	18,886.24 (20,923.67)	7771.45 (8226.37)	15,094.19 (13,570.14)	5554.49 (4881.28)	8168.21 (11,548.00)	9869.59 (12,913.69)		6567.83 (3534.17)	5193.81 (10,876.40)	2755.29 (2000.99)
***HHI***	0.05 (0.06)	0.06 (0.09)	0.17 (0.11)	0.07 (0.12)	0.06 (0.07)	0.07 (0.08)		0.05 (0.07)	0.11 (0.17)	0.10 (0.13)
***Age***	64.47 (11.07)	62.90 (12.97)	47.18 (13.72)	66.53 (13.02)	72.54 (10.69)	70.67 (11.22)		47.54 (14.26)	19.78 (28.99)	25.72 (27.21)
***Sex***										
Male	13,870 (67.62)	21,800 (50.97)	30,777 (65.45)	17,150 (46.89)	35,486 (46.57)	42,769 (53.86)		18,208 (51.53)	97,459 (54.26)	30,279 (48.48)
Female	6639 (32.37)	20,968 (49.03)	16,245 (34.55)	19,423 (53.10)	40,710 53.43	36,641 (46.14)		16,965 (48.02)	82,147 (45.73)	32,171 (51.51)
Unknown	2 (0.01)	2 (0.00)	0 (0.00)	3 (0.01)	0 (0.00)	3 (0.00)		159 (0.45)	13 (0.01)	8 (0.01)
***Health insurance***										
UEBMI	5673 (27.66)	16,478 (38.53)	5097 (10.84)	13,205 (36.10)	21,690 (28.47)	20,532 (25.85)		9243 (26.16)	22,486 (12.52)	9579 (15.34)
URBMI	8091 (39.45)	15,789 (36.92)	27,403 (58.28)	13,816 (37.77)	35,523 (46.62)	37,774 (47.57)		15,833 (44.81)	88,773 (49.42)	32,567 (52.14)
NCMS	2670 (13.02)	4827 (11.29)	9361 (19.91)	4841 (13.24)	9596 (12.59)	10,793 (13.59)		5434 (15.38)	27,017 (15.04)	9992 (16.00)
Full self-expenses	1324 (6.46)	2557 (5.98)	1526 (3.25)	2065 (5.65)	3106 (4.08)	4275 (5.38)		2712 (7.68)	25,384 (14.13)	5860 (9.38)
Other	2753 (13.42)	3119 (7.29)	3635 (7.73)	2649 (7.24)	6281 (8.24)	6039 (7.60)		2110 (5.97)	15,959 (8.88)	4460 (7.14)
***Admission source***										
Emergency	3941 (19.21)	7000 (16.37)	3854 (8.20)	6435 (17.59)	13,171 (17.29)	22,099 (27.83)		3306 (9.36)	37,693 (20.98)	9579 (15.34)
Other	16,570 (80.79)	35,770 (83.63)	43,168 (91.80)	30,141 (82.41)	63,025 (82.71)	57,314 (72.17)		32,026 (90.64)	141,926 (79.02)	52,879 (84.66)
***Urgency when admission***										
Critical urgent or Urgent	4969 (24.23)	10,070 (23.54)	3406 (7.24)	9959 (27.23)	26,884 (35.28)	32,138 (40.47)		2500 (7.08)	56,853 (31.65)	12,656 (20.26)
Common	15,542 (75.77)	32,700 (76.46)	43,616 (92.76)	26,617 (72.77)	49,312 (64.72)	47,275 (59.53)		32,832 (92.92)	122,766 (68.35)	49,802 (79.74)
***Charlson comorbidity index***	2.93 (3.72)	3.46 (3.06)	0.23 (0.65)	1.69 (1.58)	1.94 (1.72)	1.50 (1.56)		0.16 (0.57)	0.47 (1.22)	0.31 (0.81)
***Beds***	1451.91 (1206.32)	1003.99 (847.55)	719.54 (641.62)	762.67 (765.14)	817.81 (871.53)	853.84 (818.70)		680.26 (660.59)	906.93 (752.91)	613.24 (609.20)
***Hospital level***										
Primary	139 (0.68)	641 (1.50)	1345 (2.86)	1370 (3.75)	4008 (5.26)	2314 (2.91)		1883 (5.33)	3103 (1.73)	3094 (4.95)
Secondary	3657 (17.83)	10,577 (24.73)	20,245 (43.05)	12,277 (33.57)	23,614 (30.99)	27,052 (34.06)		12,260 (34.70)	55,308 (30.79)	22,148 (35.46)
Tertiary	15,683 (76.46)	27,510 (64.32)	10,819 (23.01)	17,393 (47.55)	37,340 (49.01)	41,968 (52.85)		16,587 (46.95)	109,192 (60.79)	25,689 (41.13)
Un-graded	1032 (5.03)	4042 (9.45)	14,613 (31.08)	5536 (15.14)	11,234 (14.74)	8079 (10.17)		4602 (13.03)	12,016 (6.69)	11,527 (18.46)
***Hospital ownership***										
Public	18,329 (89.36)	35,703 (83.48)	32,134 (68.34)	27,194 (74.35)	54,519 (71.55)	61,643 (77.62)		25,168 (71.23)	156,609 (87.19)	45,544 (72.92)
Private	2182 (10.64)	7067 (16.52)	14,888 (31.66)	9382 (25.65)	21,677 (28.45)	17,770 (22.38)		10,164 (28.77)	23,010 (12.81)	16,914 (27.08)
***Whether general***										
Yes	15,804 (77.05)	31,404 (73.43)	10,479 (22.29)	25,893 (70.79)	58,997 (77.43)	59,584 (75.03)		20,454 (57.89)	140,872 (78.43)	49,896 (79.89)
No	4707 (22.95)	11,366 (26.57)	36,543 (77.71)	10,683 (29.21)	17,199 (22.57)	19,829 (24.97)		14,878 (42.11)	38,747 (21.57)	12,562 (20.11)
***Whether for-profit***										
Yes	1353 (6.60)	4685 (10.95)	7077 (15.05)	5771 (15.78)	13,745 (18.04)	11,396 (14.35)		6350 (17.97)	13,410 (7.47)	11,405 (18.26)
No	19,158 (93.40)	38,085 (89.05)	39,945 (84.95)	(30,805) (84.22)	62,451 (81.96)	68,017 (85.65)		28,982 (82.03)	166,209 (92.53)	51,053 (81.74)
***Health technicians***	10.93 (6.06)	9.66 (4.97)	7.21 (3.76)	8.86 (4.65)	8.17 (4.72)	8.36 (4.66)		8.44 (4.35)	9.09 (5.25)	7.99 (4.29)
***GDP***	67,471 (32,799)	66,674 (34,509)	49,663 (24,852)	62,356 (33,848)	56,020 (32,016)	56,485 (31,169)		59,228 (32,178)	63,759 (35,661)	56,299 (30,235)
***Residences***	91.25 (48.54)	77.74 (35.82)	70.34 (28.44)	73.72 (37.18)	74.24 (34.50)	72.49 (33.02)		73.91 (33.57)	72.12 (39.24)	62.89 (34.99)
***Total***	20,511 (100.00)	42,770 (100.00)	47,022 (100.00)	36,576 (100.00)	76,196 (100.00)	79,413 (100.00)		35,332 (100.00)	179,619 (100.00)	62,458 (100.00)
**Diseases** **Variables**	**J44**	**K29**	**K80**	**M47**	**M50-M51**	**N13**	**N18**	**S06**	**S72**	**S82**
***Inpatient costs***	7515.83 (7699.11)	3622.62 (2623.34)	10,215.51 (8476.46)	4710.59 (4856.60)	5880.38 (7810.23)	7286.35 (7.976.68)	12,608.10 (24,078.80)	16,452.97 (33,588.05)	25,665.93 (22,554.44)	17,295.36 (20,273.52)
***HHI***	0.06 (0.09)	0.07 (0.11)	0.06 (0.11)	0.04 (0.06)	0.04 (0.069)	0.04 (0.072)	0.08 (0.09)	0.11 (0.15)	0.07 (0.08)	0.05 (0.08)
***Age***	72.88 (9.75)	55.44 (19.30)	54.09 (15.95)	55.92 (13.53)	57.83 (14.09)	48.75 (14.57)	57.92 (15.61)	51.27 (20.43)	69.79 (18.93)	49.61 (18.17)
***Sex***										
Male	101,115 (66.71)	25,351 (43.60)	19,553 (33.06)	21,633 (36.65)	43,530 (43.63)	24,078 (66.25)	13,565 (56.50)	15,225 (64.44)	7630 (44.24)	12,424 (56.34)
Female	50,442 (33.28)	32,775 (56.36)	39,584 (66.93)	37,391 (63.34)	56,230 (56.36)	12,266 (33.75)	10,437 (43.47)	8400 (35.55)	9616 (55.75)	9625 (43.65)
Unknown	10 (0.01)	23 (0.04)	2 (0.00)	4 (0.01)	4 (0.00)	0 (0.00)	5 (0.02)	3 (0.01)	1 (0.01)	2 (0.01)
***Health insurance***										
UEBMI	40,827 (26.94)	9933 (17.08)	13,702 (23.17)	22,641 (38.36)	31,848 (31.92)	8048 (22.14)	6607 (27.52)	1571 (6.65)	2649 (15.36)	2833 (12.85)
URBMI	70,341 (46.41)	28,883 (49.67)	25,278 (42.74)	25,068 (42.47)	42,548 (42.65)	16,325 (44.92)	10,305 (42.92)	5335 (22.58)	7044 (40.84)	7302 (33.11)
NCMS	22,763 (15.02)	11,165 (19.20)	10,223 (17.29)	6613 (11.20)	14,865 (14.90)	6036 (16.61)	2530 (10.54)	3281 (13.89)	2907 (16.86)	3293 (14.93)
Full self-expenses	5397 (3.56)	2961 (5.09)	4924 (8.33)	1914 (3.24)	3775 (3.78)	3455 (9.51)	1698 (7.07)	10,111 (42.79)	2812 (16.30)	5818 (26.38)
Other	12,239 (8.07)	5207 (8.95)	5012 (8.47)	2792 (4.73)	6728 (6.74)	2480 (6.82)	2867 (11.94)	3330 (14.09)	1835 (10.64)	2805 (12.72)
***Admission source***										
Emergency	26,491 (17.48)	7848 (13.50)	15,808 (26.73)	3595 (6.09)	8244 (8.26)	9541 (26.25)	4023 (16.76)	14,615 (61.85)	6739 (39.07)	8020 (36.37)
Other	125,076 (82.52)	50,301 (86.50)	43,331 (73.27)	55,433 (93.91)	91,520 (91.74)	26,803 (73.75)	19,984 (83.24)	9013 (38.15)	10,508 (60.93)	14,031 (63.63)
***Urgency when admission***										
Critical urgent or Urgent	56,368 (37.19)	9767 (16.80)	13,608 (23.01)	5321 (9.01)	8335 (8.35)	8183 (22.52)	6188 (25.78)	13,219 (55.95)	5177 (30.02)	5716 (25.92)
Common	95,199 (62.81)	48,382 (83.20)	45,531 (76.99)	53,707 (90.99)	91,429 (91.65)	28,161 (77.48)	17,819 (74.22)	10,409 (44.05)	12,070 (69.98)	16,335 (74.08)
***Charlson comorbidity index***	1.60 (1.50)	0.71 (1.12)	0.76 (1.28)	0.51 (0.86)	0.39 (0.80)	0.32 (0.81)	4.71 (3.37)	0.39 (0.86)	0.77 (1.17)	0.19 (0.60)
***Beds***	632.81 (658.77)	516.77 (573.87)	1025.72 (985.65)	396.01 (530.56)	445.64 (582.58)	831.62 (880.58)	1277.28 (1096.00)	938.75 (735.85)	774.95 (765.91)	661.55 (667.77)
***Hospital level***										
Primary	8778 (5.79)	4259 (7.32)	1177 (1.99)	6856 (11.61)	8562 (8.58)	2706 (7.45)	211 0.88	112 (0.47)	523 (3.03)	1052 (4.77)
Secondary	51,798 (34.17)	20,260 (34.84)	17,894 (30.26)	18,757 (31.78)	36,330 (36.42)	11,525 (31.71)	6081 25.33	7374 (31.21)	6463 (37.47)	8516 (38.62)
Tertiary	61,092 (40.31)	19,559 (33.64)	36,543 (61.79)	13,894 (23.54)	27,086 (27.15)	18,517 (50.95)	16,665 69.42	15,333 (64.89)	8944 (51.86)	9960 (45.17)
Un-graded	29,899 (19.73)	14,071 (24.20)	3525 (5.96)	19,521 (33.07)	27,786 (27.85)	3596 (9.89)	1050 4.37	809 (3.42)	1317 (7.64)	2523 (11.44)
***Hospital ownership***										
Public	101,157 (66.74)	35,954 (61.83)	49,690 (84.02)	27,209 (46.10)	51,002 (51.12)	25,572 (70.36)	20,987 (87.42)	20,438 (86.50)	13,145 (76.22)	15,486 (70.23)
Private	50,410 (33.26)	22,195 (38.17)	9449 (15.98)	31,819 (53.90)	48,762 (48.88)	10,772 (29.64)	3020 (12.58)	3190 (13.50)	4102 (23.78)	6565 (29.77)
***Whether general***										
Yes	117,883 (77.78)	43,888 (75.48)	48,121 (81.37)	35,253 (59.72)	56,247 (56.38)	28,998 (79.79)	18,716 (77.96)	19,701 (83.38)	10,528 (61.04)	12,128 (55.00)
No	33,684 (22.22)	14,261 (24.52)	11,018 (18.63)	23,775 (40.28)	43,517 (43.62)	7346 (20.21)	5291 (22.04)	3927 (16.62)	6719 (38.96)	9923 (45.00)
***Whether for-profit***										
Yes	32,853 (21.68)	16,172 (27.81)	6418 (10.85)	20,711 (35.09)	32,871 (32.95)	6896 (18.97)	2082 (8.67)	2255 (9.54)	2517 (14.59)	4197 (19.03)
No	118,714 (78.32)	41,977 (72.19)	52,721 (89.15)	38,317 (64.91)	66,893 (67.05)	29,448 (81.03)	21,925 (91.33)	21,373 (90.46)	14,730 (85.41)	17,854 (80.97)
***Health technicians***	7.641 (4.06)	6.92 (3.91)	9.02 (5.12)	8.10 (4.31)	8.10 (4.61)	8.62 (4.54)	10.14 (5.436)	8.29 (4.36)	8.29 (4.79)	8.39 (4.71)
***GDP***	57,353 (31,456)	46,818 (27,596)	62,051 (33,048)	58,205 (29,707)	56,942 (30,939)	62,699 (32,677)	64,143 (34,285)	57,230 (28,685)	56,721 (31,103)	57,591 (30,198)
***Residences***	72.83 (33.92)	61.54 (33.44)	76.78 (39.93)	72.53 (36.05)	72.42 (36.42)	73.94 (37.36)	82.84 (39.91)	70.21 (34.67)	73.77 (37.14)	73.28 (39.37)
***Total***	151,567 (100.00)	58,149 (100.00)	59,139 (100.00)	59,028 (100.00)	99,764 (100)	36,344 (100.00)	24,007 (100.00)	23,628 (100.00)	17,247 (100.00)	22,051 (100.00)

Note: (1) For continuous variables, statistics shown are the sample mean and standard deviation (in parentheses); For categorical variables, statistics shown are the frequency and percentage (in parentheses). (2) The unit of cost is Yuan. Health technicians: the number of health technicians per 1000 population. GDP: GDP per capita (Yuan). Residences: the number of residences (10,000 people). Urbanization rate: the proportion of the urban population (urbanization rate). (3) C34: bronchial and lung cancer, E11: non-insulin-dependent diabetes, F20: schizophrenia, I10: essential hypertension, I25: chronic ischemic heart disease, I63: cerebral infarction, I84: hemorrhoid, J12-J18: pneumonia, J20: acute bronchitis, J44: chronic obstructive pulmonary disease, K29: gastritis and duodenitis, K80: cholelithiasis, M47: spine joint stiffness, M50-M51: intervertebral disc disorders, N13: obstructive and reflux uropathy, N18: chronic kidney disease, S06: intracranial injury, S72: femoral fracture, S82: fractures of the lower leg (including the ankle)

The average inpatient expenses of different diseases are distinct from which femoral fracture inpatients (S72) are the highest (25,665.93 Yuan), while acute bronchitis (J20) inpatients the lowest (2755.29 Yuan). The mean values of HHI of intervertebral disc disorder (M50-M51), spine joint stiffness (M47), obstructive and reflux uropathy (N13) inpatient are the smallest (0.04), while schizophrenia (F20) inpatient the largest. The average age of pneumonia inpatients (J12–18) is the youngest (19.78), while chronic obstructive pulmonary disease (J44) inpatients the oldest (72.88). Eight diseases’ female proportion is greater than male. Most of the inpatients’ health insurance programs are the Urban Employment Basic Medical Insurance, Urban Residents Basic Medical Insurance, and New Cooperative Medical Scheme. The intracranial injury (S06) inpatient’s emergency department admission rate is the highest (61.85%), while the spine ankylosis inpatient (M47) the lowest (6.09%). More than 50% (55.95) intracranial injury (S06) inpatients’ admission status is critical or urgent, while hemorrhoids (I84) inpatients only about 7% (7.08%). The mean value of CCI of hemorrhoid (I84) inpatients is the lowest (0.16), while the non-insulin dependent diabetes (E11) the highest (3.46). Most of the inpatients are treated in secondary or tertiary hospitals, general hospitals, public hospitals, and non-profit hospitals.

### Clustering analysis

**Fig. 1:**
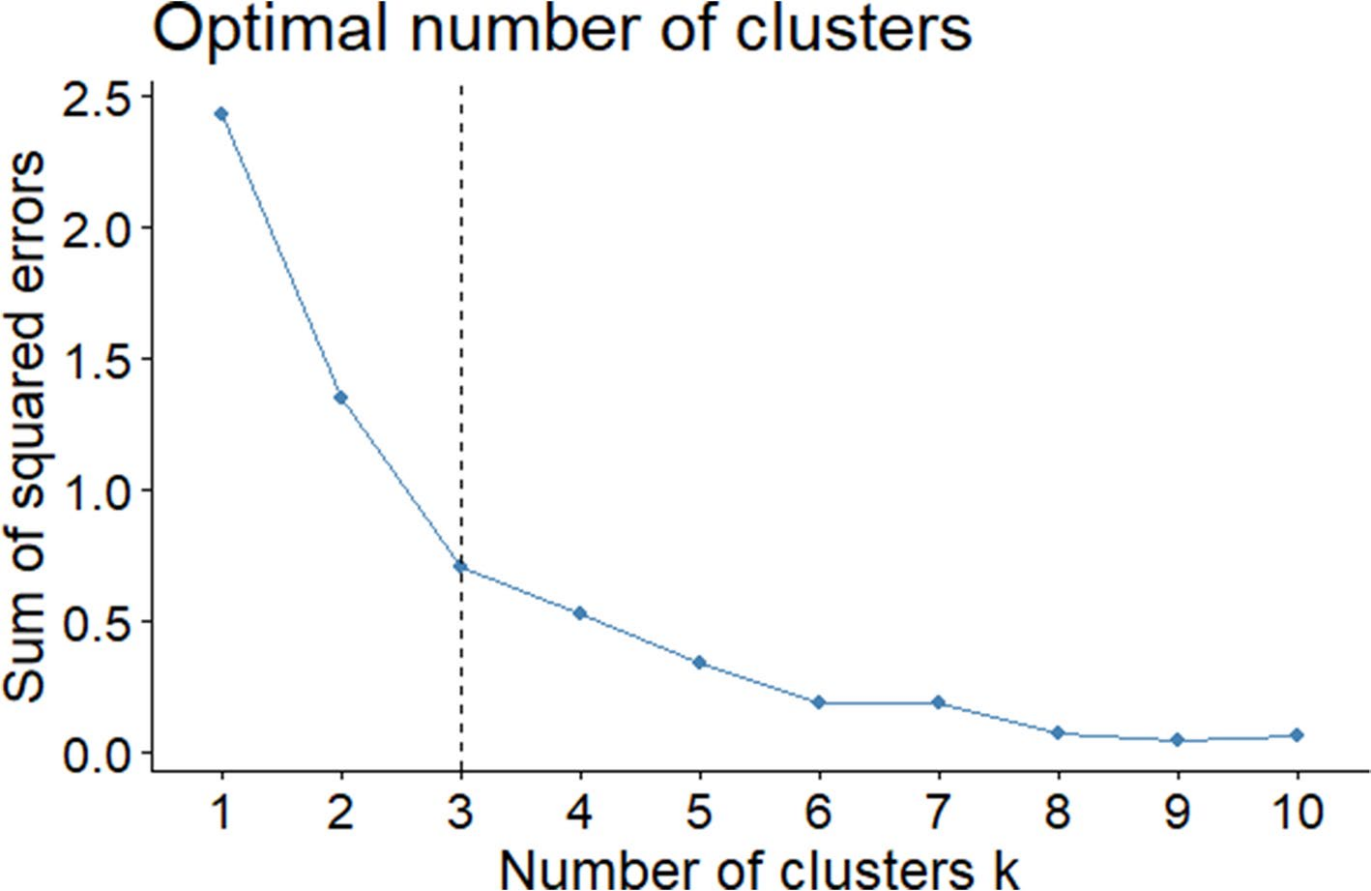
The association between the sum of squared errors and the number of clusters k

The Hopkins statistic value is 0.74 (> 0.5), suggesting that the dataset is significantly clusterable. As shown in Fig. 1, the elbow of the curve appeared when the K value is equal to 3.

Column 1 and 2 of Table 3 shows the clustering results. The bronchial and lung cancer, non-insulin-dependent diabetes, and chronic kidney disease were clustered into cluster 1. The cerebral infarction, cholelithiasis, obstructive and reflux uropathy, intracranial injury, femoral fracture, and fractures of the lower leg (including the ankle) were clustered into cluster 2. The schizophrenia, essential hypertension, chronic ischemic heart disease, hemorrhoids, pneumonia, acute bronchitis, chronic obstructive pulmonary disease, gastritis and duodenitis, spine joint stiffness, and intervertebral disc disorders were clustered into cluster 3.

**Table 3:** Clustering results

Diseases	Cluster	Groups	Emergency admission rate	Charlson Comorbidity Index
Bronchial and lung cancer (C34)	1	Disease with highly complex condition (DWC) group	0.204	0.778
Non-insulin-dependent diabetes (E11)	1			
Chronic kidney disease (N18)	1			
Cerebral infarction (I63)	2	Disease with urgent condition (DWU) group	0.543	0.108
Cholelithiasis (K80)	2			
Obstructive and reflux uropathy (N13)	2			
Intracranial injury (S06)	2			
Femoral fracture (S72)	2			
Fractures of the lower leg (including the ankle) (S82)	2			
Schizophrenia (F20)	3	Disease with less complex and less urgent (DWL) group	0.131	0.141
Essential hypertension (I10)	3			
Chronic ischemic heart disease (I25)	3			
Hemorrhoid (I84)	3			
Pneumonia (J12-J18)	3			
Acute bronchitis (J20)	3			
Chronic obstructive pulmonary disease (J44)	3			
Gastritis and duodenitis (K29)	3			
Spine joint stiffness (M47)	3			
Intervertebral disc disorders (M50-M51)	3			

Column 3, 4, and 5 of Table 3 shows the centroid point of each cluster, which could reflect the characteristics of each cluster (all clustering indicators were scaled before clustering). Cluster 1 refers to diseases with highly complex condition (DWC) group characterized by high CCI. Cluster 2 refers to diseases with urgent condition (DWU) group characterized by high emergency admission rates. Cluster 3 refers to diseases with less complex and less urgent condition (DWL) group characterized by low CCI and low emergency admission rates.

To verify whether the indicators used for clustering contribute to the results of the clustering process, the Kruskal-Wallis test was conducted in this study. The results show that the *P*-value of the cluster indicators in the three clusters are all < 0.05, suggesting that the difference in the three clusters is statistically significant. Therefore, the two indicators were used for cluster analysis of 19 diseases, and it is reasonable to use them as the indicators for cluster analysis.

### Regression analysis

**Fig. 2:**
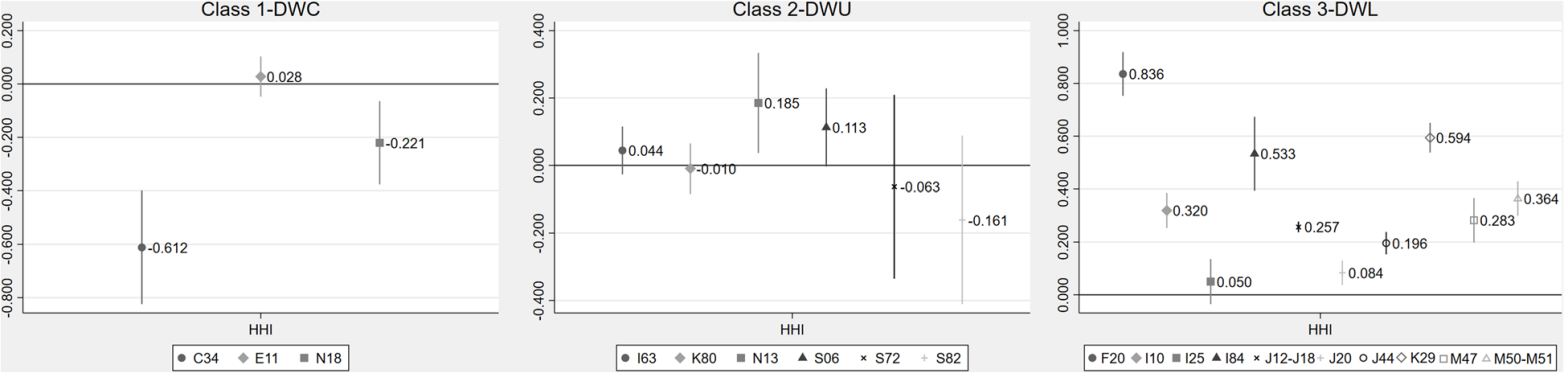
**Regression analysis.** Note: (1) DWC group refers to diseases with highly complex conditions. DWU group refers to diseases with urgent conditions. DWL group refers to diseases with less complex and urgent conditions. (2) C34: bronchial and lung cancer. E11: non-insulin-dependent diabetes. N18: chronic kidney disease. (3) I63: cerebral infarction. K80: cholelithiasis. N13: obstructive and reflux uropathy. S06: intracranial injury. S72: femoral fracture. S82: fractures of the lower leg (including the ankle). (4) F20: schizophrenia. I10: essential hypertension. I25: chronic ischemic heart disease. I84: hemorrhoids. J12-J18: pneumonia. J20: acute bronchitis. J44: chronic obstructive pulmonary disease. K29: gastritis and duodenitis. M47: spine joint stiffness. M50-M51: intervertebral disc disorders

Figure 2 shows the estimated coefficient of HHI for all diseases and its 95% confidence interval.^8^

For diseases with highly complex conditions, the estimated coefficients of HHI are mixed in the direction and statistical significance in the identical regression model. For bronchial and lung cancer (C34) and chronic kidney disease (N18), the coefficients of HHI are − 0.612 and − 0.221 respectively, and both statistically significant, which means that these diseases’ inpatient expenses increase by 6.12 and 2.21% respectively, on average with 10% reduction in HHI. For non-insulin-dependent diabetes (E11), the coefficient of HHI is 0.028, and statistically insignificant at 5% level, indicating that there are no effects of hospital competition on inpatient expenses of this disease.

For diseases with urgent conditions, the estimated coefficients of HHI are also mixed in the direction and statistical significance in the identical regression model (almost all of them are statistically insignificant at 5% level). For cerebral infarction (I63), cholelithiasis (K80), intracranial injury (S06), femoral fracture (S72), and fractures of the lower leg (including the ankle) (S82), the coefficients of HHI are − 0.044, − 0.010, 0.113, − 0.063, and − 0.161, respectively, and all statistically insignificant at 5% level, indicating that there are no effects of hospital competition on inpatient expenses of these three diseases. For obstructive and reflux uropathy (N13), the coefficient of HHI is 0.185, and significant at 5% level, suggesting that the inpatient expenses decrease by 1.85% on average with 10% reduction in HHI.

For diseases with less complex and less urgent conditions, the coefficients of HHI are all positive, and almost all of them significant at the 5% level. Specifically, the coefficients of HHI of schizophrenia (F20), essential hypertension (I10), hemorrhoids (I84), pneumonia (J12-J18), acute bronchitis (J20), chronic obstructive pulmonary disease (J44), gastritis and duodenitis (K29), spine joint stiffness (M47), and intervertebral disc disorders (M50-M51) are 0.836, 0.320, 0.533, 0.257, 0.084, 0.196, 0.594, 0.283, and 0.364 respectively, and all significant at the 5% level, which shows that when HHI is reduced by 10% on average, the inpatient expenses of these diseases are reduced by 8.36, 3.20, 5.33, 2.57, 0.84, 1.96, 5.94, 2.83, and 3.64% on average respectively. The results suggest that hospital competition would reduce these diseases’ inpatient expenses. The coefficient of HHI of the chronic ischemic heart disease (I25) is 0.050, and insignificant at 5% level, indicating that there are no effects of hospital competition on inpatient expenses of this disease.

In summary, the results suggest that heterogeneous effects of hospital competition on inpatient expenses across disease groups: hospital competition does not play an ideal role in reducing inpatient expenses for diseases with highly complex conditions and diseases with urgent conditions, but it has a significant effect on reducing inpatient expenses of diseases with less complex and less urgent conditions.

### Robust check

We used 3 digits ICD-10 code to identify the observations in this study. Only using 3-digits ICD-10-codes may make the categories overly broad in terms of complexity and acuteness. We also controlled a set of dummy variables of the complete ICD-10 code of primary diagnosis in the regression model to control the fixed effects of diseases furtherly, the findings are consistent with our main analysis. Appendix Fig. 2 in the supplementary file shows the results controlling a set of dummy variables of the complete ICD-10 code of primary diagnosis.

In the predicted patient flow approach, inpatients’ choice set ***J*** was restricted to their chosen hospital and all hospitals within 100 km. To test the robustness of our results, this study also used different lengths to define the inpatients’ choice set ***J***, including 80 km, 150 km, and 200 km. Using the identical regression model as the main regression analysis, the results are similar to the main regression analysis, showing the robustness of our results. The result is shown in Appendix Fig. 3, 4, 5 in the supplementary file.

We conducted a series of tests before and after clustering to ensure and verify the accuracy of clustering results as much as possible. In addition, to avoid the potential bias caused by the K-means clustering method and test the robustness of our results, instead of grouping the selected diseases by the K-means clustering method, we directly added the disease-specific emergency admission rate and the disease-specific average CCI into the regression model as proxy variables for the urgency and complexity of diseases condition, and construct interaction terms with HHI to verify whether the heterogeneity of competition still exists. The results are displayed in Appendix Table 6. We also divided the disease-specific emergency admission rate and the disease-specific average CCI into three groups according to the quantile, and also construct interaction terms with HHI to verify whether the heterogeneity of competition still exists. The results are displayed in Appendix Table 7. The findings are still consistent with the main analysis, namely, the more complex and urgent the disease, the weaker the role of hospital competition in reducing inpatient expenses, and even increase expenses.

## Discussion

This study selected 19 representative diseases with significant burden based on the inpatient volume and expenses and explored the heterogeneous effects of hospital competition on inpatient expenses basing on disease grouping according to condition’s complexity and urgency. Employing K-means clustering, the selected diseases were divided into three groups, including diseases with highly complex conditions, diseases with urgent conditions, and diseases with less complex and less urgent conditions. Calculating Herfindahl-Hirschman Index based on the predicted patient flow to measure the hospital competition, and using the log-linear multivariate regression model, we found heterogeneous effects of hospital competition on inpatient expenses across disease groups: hospital competition does not play an ideal role in reducing inpatient expenses for diseases with highly complex conditions and diseases with urgent conditions, but it has a significant effect in reducing inpatient expenses of diseases with less complex and less urgent conditions.

The difficulties of obtaining or understanding the disease- and treatment-related knowledge for patients who suffer from diseases with highly complex conditions would be bigger than their counterparts, which would lead to these patients facing greater information asymmetry. These patients in the process of care provision usually cannot know what treatment or service options are desirable or necessary for their specific medical condition. Since the major revenue of hospitals comes from patient fees [42] 9, hospitals facing huge competitive pressures would have incentives to provide more services for patients to earn more revenue. The hospitals in competitive market may not attract these patients with highly complex conditions by improving the efficiency to decrease the expenses as the providers in the general market but induce demand by using information asymmetry between physicians and patients or achieve the purpose of profit maximization by carrying out medical arms race, mixing the effectiveness of hospital competition in reducing the inpatient expenses of diseases with highly complex conditions [43–45].

The choice autonomy for the patients who suffer from the disease with urgent conditions would be limited greatly. These patients usually need to be admitted to the hospital for treatment within a very short time, generally selecting the nearest hospital according to treatment capacity [21]. Even in some emergencies, patients or their companions have to dial the emergency phone for medical help. In this case, the ambulance drivers are usually instructed to bring patients to the nearest emergency room [46], which would leave the patients or their companion limited opportunity to choose hospitals. The choice autonomy for these patients would be limited greatly, affecting the effectiveness of hospital competition on inpatient expenses [19, 20]. In addition, the patients suffering from the disease with urgent conditions typically exhibit a very low elasticity of demand for hospital treatment [21]. Since the distance from the patient to the hospital would be the main consideration of these patients, they would not be attracted dramatically by the hospital’s competitive behaviors, such as reducing medical expenses. As a result, the hospitals have not great incentives to reduce expenses to attract these patients, leading to the hospital competition insignificantly associated with inpatient expenses of diseases with urgent conditions.

The information asymmetry and patient’s limited choice autonomy in the process of care provision for the patients who suffer from diseases with less complex and less urgent conditions would not be obvious. Patients with these diseases can obtain information and knowledge about diseases or the treatment by searching the internet or asking their friends who have suffered from, helping them to judge to a certain extent what treatment or service options are desirable or necessary for their specific medical condition in the process of care provision. The information gap between physicians and patients would be reduced, decreasing the possibility that the hospitals adopt the strategies for their own benefits at the sacrifice of compromising patients’ interests. These diseases’ condition is less urgent, and the patients or their companion would have enough time to choose the hospital to maximize their utility. Compared with patients who suffer from the disease with urgent conditions, patients with these diseases exhibit higher elasticity of demand for hospital treatment or services. These patients would be more sensitive to the medical expenses than their counterparts, leading to the hospitals in competitive market would take measures, including reducing the inpatient expenses, to attract patients to obtain the competitive advantage. The care of diseases with less complex and less urgent conditions is more similar to the product in the general market, and the hospital competition could play a positive role in reducing the inpatient expenses.

The findings of this study are consistent with previous studies focusing on diseases with a specific condition from the United States [47, 48], and China [19]. Using the patients undergoing hepatic or pancreatic resection as the representative of highly complex and specialized surgery procedures, M Cerullo, et al. [47] examined the association between regional hospital market concentration and hospital charges for hepatopancreatic biliary surgical procedures. This study concluded that for complex, highly specialized procedures, hospital competition would negatively associate with overall charges. OY Tang, et al. [48] evaluated the relationship between interhospital competition and inpatient charges in patients undergoing cranial neurosurgery that is usually complex and found that hospitals in more competitive markets exhibited higher charges for admissions of patients undergoing an in-hospital cranial procedure. Both of their findings are consistent with our study’s finding that hospital competition does not play an ideal role in reducing inpatient expenses for diseases with highly complex conditions. C Deng and J Pan [19] evaluated and compared the relationships between hospital competition and the expenses of prostatectomies (elective surgery, representing treatments of non-acute common diseases) and appendectomies (emergency surgery, representing treatments of acute common diseases), and found that greater competition was significantly associated with lower total hospital charges for prostatectomies, while the opposite was true for appendectomies, which are consistent with our study, namely, hospital competition does not play an ideal role in reducing inpatient expenses for diseases with urgent conditions, while it has a significant effect in reducing inpatient expenses of diseases with less complex and less urgent conditions.

Taking into consideration multiple factors as discussed above, it is therefore highlighted as an essential implication in our study that the differences in condition’s complexity and urgency among diseases would lead to different impacts of hospital competition. When using hospital competition to achieve the optimal policy impact on healthcare systems while minimizing the potential adverse outcomes to improve the health services supply systems, the differences in conditions among diseases should be given full consideration. The authority should pay more attention to the diseases with highly complex and urgent conditions to reduce the potential negative influence caused by information asymmetry and limited choice autonomy.

It is noteworthy that some limitations should be addressed in this study. (1) This study collected data from the discharge data of inpatients of Sichuan province in the fourth quarter of 2018 (from September to December), there may be a potential seasonal trend. In future research, we can collect more longitudinal data to solve this problem. (2) The sample size only covers hospitals in Sichuan province. Although Sichuan province can reflect the overall situation of the country to a certain degree, for some specific regions, such as Shanghai and Shenzhen cities, the generalizability of our findings would be limited. More data collected from several provinces should be used to analyze furtherly in future studies. (3) Research focusing on the mechanisms of competition effects is needed. (4) This study used K-means clustering based on objective clustering indicators to group the diseases. Due to the data limitation, the selected indicators may not fully reflect the conditions’ complexity and urgency of diseases. More systematic and comprehensive indicators should be collected and selected as the cluster indicators to fully reflect disease conditions’ complexity and urgency in future studies.

## Conclusion

Based on the results of K-means clustering and log-linear multivariate regression model, we concluded that heterogeneous effects of hospital competition on inpatient expenses across diseases groups: hospital competition does not play an ideal role in reducing inpatient expenses for diseases with highly complex conditions and diseases with urgent conditions, but it has a significant effect in reducing inpatient expenses of diseases with less complex and less urgent conditions. Our study offers implications that the differences in conditions’ complexity and urgency among diseases would lead to different impacts of hospital competition, which would be given full consideration when designing the pro-competition policy in the healthcare delivery system to achieve the desired goal.

## Supplementary Information

**Additional file 1.**

## Data Availability

The data that support the findings of this study are available from the Health Commission of Sichuan Province but restrictions apply to the availability of these data, which were used under license for the current study, and so are not publicly available. Data are however available from the authors upon reasonable request and with permission of the Health Commission of Sichuan Province.

## Abbreviations

HHI: Herfindahl-Hirschman Index
ICD-10: International Statistical Classification of Diseases and Related Health Problems, 10th Revision
DWC: Diseases with highly complex condition group
DWU: Diseases with urgent condition group
DWL: Diseases with less complex and less urgent condition group
CCI: Charlson comorbidity index (CCI)
M: Means
SDs: Standard deviation

## Footnotes

1 The representativeness of Sichuan Province is discussed in the appendix text (Text 1) in the supplementary file.

2 Although senile cataract (H25) also meets the above inclusion criteria, there are many special subsidy policies for the patients with H25, such as reduction or exemption of operation expenses for the rural elderly poor, which would likely impact the effects of competition on the medical expenses. We excluded this disease in our study to avoid its confounding effects caused by the policies on our analysis [29–31].

3 The results of the choice model of all diseases are displayed in Appendix Table 1 in the supplementary file.

4 A detailed explanation of how to construct the HHI based on the predicted patient flow approach is shown in the appendix text (Text 2) in the supplementary file.

5 A value of Hopkins statistic is close to 1 (far above 0.5), suggesting that the dataset is significantly clusterable. A value around 0.5 indicates that the data is random, and uniformly distributed data will tend to result in values close to 0 [37, 38].

6 The elbow method is used to determine the number of clusters (K). This method consists of plotting the explained variation as a function of the number of clusters and picking the elbow of the curve as the optimal number of clusters [39].

7 Appendix Table 2 in the supplementary file displays the number and percentage of all kinds of hospitals for each disease.

8 The regression results of all variables are displayed in Appendix Table 3 (DWC group), 4 (DWU group), and 5 (DWL group) in the supplementary file.

9 In China, only about 10% of the hospital’s revenue comes from government subsidies, and the vast shortfall needs to be filled by patient fees [42].
