# Supplementary material for: Heterogeneous effects of hospital competition on inpatient expenses: an empirical analysis of diseases grouping basing on conditions’ complexity and urgency
Source: BMC Health Serv Res. 2021 Dec 10;21:1322. doi: 10.1186/s12913-021-07331-1 (PMC8662870; doi:10.1186/s12913-021-07331-1)
Supplement: Supplementary file 1 — Additional file 1. [file 12913_2021_7331_MOESM1_ESM.docx]

**Article title**

Heterogeneous effects of hospital competition on inpatient expenses: An empirical analysis of diseases grouping basing on conditions’ complexity and urgency

**Journal name**

BMC Health Services Research

**Contact information of authors**

Liyong Lu^1,2^, Xiaojun Lin^1,2^, Jay Pan^1,2^

^1^HEOA Group, West China School of Public Health and West China Fourth Hospital, Sichuan University

^2^Institute for Healthy Cities and West China Research Center for Rural Health Development, Sichuan University

**Content**

[Appendix text: 3](#_Toc86002074)

[Text 1: The discussion of representativeness of Sichuan Province 3](#_Toc86002075)

[Text 2: A detailed explanation of how to construct the HHI based on the predicted patient flow approach 4](#_Toc86002076)

[Text 3: A detailed explanation of why we use disease-specific inpatient emergency admission rate and CCI as the clustering indicators 7](#_Toc86002077)

[Appendix Tables: 9](#_Toc86002078)

[Appendix Table 1 Results of choice model 9](#_Toc86002079)

[Continued Appendix Table 1 Results of choice model 11](#_Toc86002080)

[Appendix Table 2 Number and percentage of all kinds of hospitals for each disease 14](#_Toc86002081)

[Continued Appendix Table 2 Number and percentage of all kinds of hospitals for each disease 14](#_Toc86002082)

[Appendix Table 3 Regression results of highly complex condition (DWC) group 16](#_Toc86002083)

[Appendix Table 4 Regression results of urgent condition (DWU) group 18](#_Toc86002084)

[Appendix Table 5 Regression results of less complex and less urgent condition (DWL) group 20](#_Toc86002085)

[Appendix Table 6 Regression results adding interaction terms (continuous variables) 23](#_Toc86002086)

[Appendix Table 7 Regression results adding interaction terms (categorical variables) 24](#_Toc86002087)

[Appendix Figures: 25](#_Toc86002088)

[Appendix Figure 1 Regression analysis controlling LOS and inpatient mortality 25](#_Toc86002089)

[Appendix Figure 2 Regression analysis controlling a set of dummy variables of the complete ICD-10 code of primary diagnosis 26](#_Toc86002090)

[Appendix Figure 3 Regression analysis: 80km 27](#_Toc86002091)

[Appendix Figure 4 Regression analysis: 150km 28](#_Toc86002092)

[Appendix Figure 5 Regression analysis: 200km 29](#_Toc86002093)

[Reference 30](#_Toc86002094)

Appendix text:

**Text 1: The discussion of representativeness of Sichuan Province**

Sichuan province would present a miniature of China. The Hu, Huanyong Line (Hu Line), also called the Heihe-Tengchong Line proposed in 1935 by Hu, Huanyong marks a striking difference in the distribution of China's population. 36% of China's total land area is covered by plains and 96% of its population are distributed in the area of China to the southeast of the Hu Line, while 64% of China's land area is covered by mountains but only 4% of its population in the area to the northwest of the Hu Line[1]. Sichuan Province was selected as a case study for this research as it provides a scaled-down model of the broader situation in China. First, Hu Line is across Sichuan and separated into two cultural/topographic sections. The main terrains of the southeast section of Sichuan are the productive plains and hills which support the majority of the population and a well-developed economy, while the mountain area in the northwest section of the province is sparsely populated with low economic levels. The differences in the distribution of topography, economy, and population among regions would lead to large diversities in the local healthcare development as well as the healthcare market. Most medical resources and healthcare facilities are distributed in the east area of Sichuan province, and sparsely in the west area of Sichuan province, which is also similar to the condition of the whole county to a certain degree [2, 3].

**Text 2: A detailed explanation of how to construct the HHI based on the predicted patient flow approach**

Specifically, the process to measure the hospital competition by predicted patient flow method are as follows [4-7]:

First, we constructed the patient’s hospital choice model. Consider an inpatient *i* who chooses among the ***J*** hospitals in her area. ***J*** may vary across individuals, and denotes the patient's potential hospital choice sets. According to the previous study, ***J*** was restricted to their chosen hospital and all hospitals within 100 km [4-6] (we also used different lengths to define the inpatients’ choice set ***J*** in the robust test). The *j_th_* hospital (*j* = 1, . . ., *J*) has *k* binary characteristics describing its level, size, and ownership status, denoted by *Z_j_^1^*, *Z_j_^2^*, *Z_j_^3^*.

The hospital choice of inpatient *i*’s depends on the utility from that choice, and inpatient utility from choosing hospital *j* was determined directly by the inpatient’s and hospital *j*’s characteristics [4-7]. In addition, since the distance between inpatient *i* and hospital *j* reflects the travel costs, it is also an important determinant of hospital choice decisions for inpatients. This study hypothesizes that choice decision between any two hospitals is not independent. The relative utility for inpatient *i* of choosing one particular hospital *j* versus another *j'* depends not only on the characteristics of *j* and *j'*, but also on the characteristics of other hospitals that may be good or poor substitutes for *j* and *j'* [4]. Therefore, in addition to inpatient’s and hospital *j*’s characteristics, the inpatient utility from choosing hospital *j* was also determined by the distance of *i* to *j* relative to the distance of *i* to the nearest hospital that is either a good substitute or a poor substitute for *j* in characteristics *Z_j_^1^*, *Z_j_^2^*, *Z_j_^3^* [4]. According to the above discussion, our model hypothesizes that inpatient *i*’s hospital choice depends on inpatient’s utility from that choice, and inpatient’s utility from choosing hospital *j* not only depends on inpatient’s and hospital *j*’s characteristics but also the distance of inpatient *i* to hospital *j* relative to the distance of *i* to the nearest hospital that is either a good substitute or a poor substitute for *j* in characteristics *Z_j_^1^*, *Z_j_^2^*, *Z_j_^3^* [4]. The choice model is as follow:

| $U_{ij}=\sum_{k=1}^{3} \left\{\begin{matrix} \beta_{1}^{k}\left(d_{ij}-d_{ij^{+}}^{k}\right)\times z_{j}^{k}+\beta_{2}^{k}\left(d_{ij}-d_{ij^{+}}^{k}\right)\times\left(1-z_{j}^{k}\right) \\ \text{+}\beta_{3}^{k}\left(d_{ij}-d_{ij^{-}}^{k}\right)\times z_{j}^{k}+\beta_{4}^{k}\left(d_{ij}-d_{ij^{-}}^{k}\right)\times\left(1-z_{j}^{k}\right) \\ \text{+}\beta_{5}^{k}(female_{i}\times z_{j}^{k}) \\ +\beta_{6}^{k}(age_{i}\times z_{j}^{k}) \\ +\beta_{7}^{k}(highseverity_{i}\times z_{j}^{k}) \\ +\beta_{8}^{k}(\text{s}erious_{i}\times z_{j}^{k}) \\ +\beta_{9}^{k}(emergency_{i}\times z_{j}^{k}) \\ +\beta_{10}^{k}(CCI_{i}\times z_{j}^{k}) \end{matrix}\right\}+e_{ij}$ | (A1) |
| --- | --- |

Where $U_{ij}$ denotes inpatient *i*’s indirect expected utility from choosing hospital *j*, as the sum of a function of the relative distances and hospital characteristics *Z_j_^1^*, *Z_j_^2^*, *Z_j_^3^*; a function of inpatient *i*’s demographic characteristics *female_i_*, *age_i_*, *lowseverity_i_*, *emergency_i_* and *CCI_i_* and hospital characteristics *Z_j_^1^*, *Z_j_^2^*, *Z_j_^3^*; and random error$e_{ij}$. Among them, *Z_j_^1^*, *Z_j_^2^*, *Z_j_^3^* represents whether hospital *j* is a public hospital, whether hospital *j* is a tertiary hospital, whether hospital *j* is a big hospital (defined as the actual number of hospital beds over the median bed for a specific disease), respectively; *female_i_* and *age_i_* denote the gender and age of inpatient *i*, respectively. *highseverity_i_* is binary indicator of whether inpatient *i* has more than three diagnosis codes in their secondary diagnoses (then low severity as the reference group). *serious_i_* is binary indicator of the inpatient *i*’s urgency at admission is critical urgent or urgent (general as the reference group). *emergency_i_* indicates whether inpatient *i* is admitted through the emergency department (admission not from the emergency department as the reference group). *CCI_i_* denotes Charlson Comorbidity Index (CCI) to reflect the complications of inpatients.

We further explain the first four terms of formula (1). The first four terms of formula (1) parameterize the utility of inpatient *i* from choosing hospital *j* as a function of relative distances: relative distances that depend on the location of hospitals that are good or poor substitutes for *j* (same- or different-type relative distances in terms of characteristics *Z_j_^1^*, *Z_j_^2^*, *Z_j_^3^*). Specifically, $d_{ij}-d_{ij^{+}}^{k}$ is the distance from inpatient *i*’s residence to hospital *j* minus the distance from *i*’s residence to the nearest hospital with the same characteristics *Z_j_^1^*, *Z_j_^2^*, *Z_j_^3^*. When $d_{ij}-d_{ij^{+}}^{k}$ >> 0, it means that the availability at low travel cost of good substitutes for hospital *j* in one or more dimensions, which may reduce inpatient *i*’s utility from choosing hospital *j*. Second, $d_{ij}-d_{ij^{-}}^{k}$is the distance from *i*’s residence to hospital *j* minus the distance from *i*’s residence to the nearest hospital with different characteristics in terms of *Z_j_^1^*, *Z_j_^2^*, *Z_j_^3^*. Since the availability at travel cost of poor substitutes for hospital *j* in one or more dimensions may also affect *i*’s utility from choosing *j*, $d_{ij}-d_{ij^{-}}^{k}$ was also included in the choice model [4, 5].

Second, we predicted the probability of inpatient *i* admitted to hospital *j* in their choice set ***J*** based on the above hospital choice model.

|  | (A2) |
| --- | --- |

whereis the utility of inpatient *i* admitting to hospital *j*.

Third, the HHI for inpatient *i* was calculated.

|  | (A3) |
| --- | --- |

Fourth, HHI for hospital *j* was calculated.

|  | (A4) |
| --- | --- |

where *I* refers to these inpatients who might potentially choose hospital *j*, indicates the expected volume of hospital *j*.

**Text 3: A detailed explanation of why we use disease-specific inpatient emergency admission rate and CCI as the clustering indicators**

According to the aims of this study, the clustering indicators were selected from the point of complexity and urgency of the disease condition.

This study considered whether the disease conditions are urgent rather than acute. Acute disease conditions do not mean that the condition is urgent. For example, although acute bronchitis is a kind of acute disease, the patients usually are less urgent and have time to select the hospital. The patients admitting from the emergency department generally mean unplanned admission, and their disease conditions are urgent, leaving them or their companions limited choice to select hospitals [8]. This study used the disease-specific inpatient emergency admission rate to measure the urgency of the disease conditions. In addition, using the admission urgency of inpatients, we also calculated the disease-specific inpatient urgent rate of admission to reflect the urgency of the disease conditions.

Since the complication could reflect the complexity of the disease conditions to some extent [9], we calculated the disease-specific inpatient average Charlson Comorbidity Index (CCI) to reflect the complexity of the disease conditions. In addition, the disease-specific inpatient average number of secondary diagnoses was also used to measure the complexity [10].

Before the clustering analysis, we calculated the Pearson correlation coefficient matrix to measure the similarity of cluster indicators [9, 11]. The Pearson correlation coefficient is close to 1 or - 1. When the Pearson correlation coefficient is greater than 0.70, the correlation between two indicators is highly strong, suggesting that two indicators can be substituted for each other [12].

The results show that the Pearson correlation coefficient between the disease-specific inpatient emergency admission rate and the urgent rate of admission is 0.78, and between the disease-specific inpatient average CCI and number of secondary diagnoses is 0.87, suggesting that the disease-specific inpatient urgent rate of admission and average number of secondary diagnoses can be substituted by the disease-specific inpatient emergency admission rate and the average CCI, respectively. Therefore, we excluded the disease-specific inpatient urgent rate of admission and average number of secondary diagnoses in the clustering analysis, and the selected clustering indicators include the disease-specific inpatient emergency admission rate and the average CCI.

Appendix Tables:

**Appendix Table 1 Results of choice model**

| Variables | C34 | E11 | F20 | I10 | I25 | I63 | I84 | J12-J18 | J20 | J44 |
| --- | --- | --- | --- | --- | --- | --- | --- | --- | --- | --- |
| dd_goodsub_z^1^*z^1^ | 0.007 | -0.069*** | 0.002 | -0.034*** | -0.036*** | -0.044*** | -0.012*** | -0.053*** | -0.053*** | -0.043*** |
|  | (0.005) | (0.003) | (0.001) | (0.003) | (0.002) | (0.002) | (0.002) | (0.001) | (0.001) | (0.001) |
| dd_goodsub_z^1^*(1-z^1^) | 0.027*** | 0.032*** | 0.034*** | 0.005 | -0.023*** | -0.035*** | -0.032*** | 0.040*** | 0.032*** | 0.017*** |
|  | (0.006) | (0.005) | (0.002) | (0.004) | (0.003) | (0.003) | (0.003) | (0.003) | (0.002) | (0.002) |
| dd_poorsub_z^1^*z^1^ | 0.039*** | 0.050*** | 0.066*** | 0.006 | -0.028*** | -0.038*** | -0.037*** | 0.071*** | 0.051*** | 0.013*** |
|  | (0.006) | (0.005) | (0.002) | (0.004) | (0.003) | (0.004) | (0.004) | (0.003) | (0.003) | (0.003) |
| dd_poorsub_z^1^*(1-z^1^) | 0.039*** | -0.057*** | 0.012*** | -0.035*** | -0.029*** | -0.034*** | 0.017*** | -0.035*** | -0.046*** | -0.044*** |
|  | (0.006) | (0.004) | (0.002) | (0.004) | (0.003) | (0.003) | (0.004) | (0.002) | (0.002) | (0.002) |
| dd_goodsub_z^2^*z^2^ | -0.091*** | -0.089*** | 0.002** | -0.041*** | -0.064*** | -0.059*** | -0.033*** | -0.082*** | -0.053*** | -0.050*** |
|  | (0.002) | (0.001) | (0.001) | (0.001) | (0.001) | (0.001) | (0.001) | (0.001) | (0.001) | (0.001) |
| dd_goodsub_z^2^*(1-z^2^) | -0.001 | 0.008 | -0.052*** | -0.027*** | -0.020*** | -0.041*** | -0.011** | -0.054*** | -0.066*** | -0.078*** |
|  | (0.005) | (0.005) | (0.002) | (0.004) | (0.004) | (0.003) | (0.004) | (0.002) | (0.002) | (0.003) |
| dd_poorsub_z^2^*z^2^ | 0.023*** | 0.033*** | -0.044*** | -0.006 | 0.016*** | -0.021*** | 0.028*** | -0.025*** | -0.042*** | -0.050*** |
|  | (0.006) | (0.005) | (0.002) | (0.004) | (0.004) | (0.004) | (0.004) | (0.003) | (0.003) | (0.003) |
| dd_poorsub_z^2^*(1-z^2^) | -0.121*** | -0.115*** | -0.007*** | -0.061*** | -0.095*** | -0.085*** | -0.044*** | -0.105*** | -0.072*** | -0.065*** |
|  | (0.002) | (0.002) | (0.001) | (0.002) | (0.001) | (0.001) | (0.002) | (0.001) | (0.001) | (0.001) |
| dd_goodsub_z^3^*z^3^ | -0.022*** | 0.027*** | -0.029*** | 0.010*** | 0.045*** | 0.014*** | 0.017*** | -0.013*** | 0.021*** | 0.030*** |
|  | (0.003) | (0.003) | (0.001) | (0.002) | (0.003) | (0.002) | (0.003) | (0.001) | (0.001) | (0.001) |
| dd_goodsub_z^3^*(1-z^3^) | 0.018** | 0.025*** | 0.003* | 0.037*** | 0.028*** | 0.120*** | 0.015*** | 0.055*** | 0.042*** | 0.043*** |
|  | (0.006) | (0.005) | (0.002) | (0.005) | (0.003) | (0.005) | (0.003) | (0.003) | (0.002) | (0.002) |
| dd_poorsub_z^3^*z^3^ | 0.044*** | 0.036*** | -0.031*** | 0.038*** | 0.043*** | 0.120*** | 0.008 | 0.070*** | 0.040*** | 0.058*** |
|  | (0.006) | (0.006) | (0.002) | (0.005) | (0.004) | (0.005) | (0.004) | (0.003) | (0.003) | (0.002) |
| dd_poorsub_z^3^*(1-z^3^) | -0.026*** | 0.048*** | -0.028*** | 0.031*** | 0.083*** | 0.019*** | 0.013** | -0.007*** | 0.040*** | 0.065*** |
|  | (0.005) | (0.005) | (0.002) | (0.004) | (0.003) | (0.004) | (0.004) | (0.002) | (0.002) | (0.002) |
| ref=male*z^1^ |  |  |  |  |  |  |  |  |  |  |
| female*z^1^ | 0.189** | 0.103** | -0.097*** | -0.011 | 0.044 | 0.043 | 0.149*** | 0.671*** | 0.676*** | 0.128*** |
|  | (0.061) | (0.037) | (0.027) | (0.033) | (0.023) | (0.023) | (0.034) | (0.015) | (0.023) | (0.016) |
| age*z^1^ | 0.014*** | 0.003*** | -0.007*** | 0.006*** | 0.008*** | 0.012*** | 0.027*** | -0.004*** | -0.008*** | 0.008*** |
|  | (0.001) | (0.001) | (0.000) | (0.001) | (0.000) | (0.000) | (0.001) | (0.000) | (0.001) | (0.000) |
| high severity*z^1^ | -0.012 | 0.173*** | 0.430*** | 0.036 | 0.123*** | -0.114*** | -0.574*** | -0.016 | -0.345*** | -0.086*** |
|  | (0.067) | (0.049) | (0.055) | (0.042) | (0.033) | (0.030) | (0.042) | (0.031) | (0.040) | (0.019) |
| serious*z^1^ | 0.356*** | 0.051 | 0.450*** | -0.123** | 0.161*** | 0.218*** | 0.687*** | 0.661*** | 0.685*** | 0.199*** |
|  | (0.069) | (0.049) | (0.060) | (0.040) | (0.025) | (0.025) | (0.076) | (0.022) | (0.036) | (0.016) |
| emergency*z^1^ | -0.250*** | 0.738*** | -0.047 | 0.667*** | 1.074*** | 0.781*** | 0.299*** | 0.515*** | 1.149*** | 0.872*** |
|  | (0.073) | (0.059) | (0.057) | (0.049) | (0.038) | (0.032) | (0.056) | (0.025) | (0.046) | (0.024) |
| CCI*z^1^ | -0.018* | 0.013 | 0.208*** | 0.031** | -0.024** | -0.092*** | -0.294*** | -0.022* | 0.049** | -0.013* |
|  | (0.009) | (0.008) | (0.031) | (0.011) | (0.007) | (0.008) | (0.030) | (0.009) | (0.017) | (0.006) |
| female*z^2^ | 0.113* | -0.077** | 0.209*** | 0.083** | -0.214*** | -0.089*** | 0.082** | -0.058*** | 0.036 | -0.076*** |
|  | (0.047) | (0.028) | (0.030) | (0.027) | (0.020) | (0.019) | (0.027) | (0.012) | (0.021) | (0.015) |
| age*z^2^ | -0.005*** | -0.006*** | -0.033*** | -0.004*** | -0.007*** | -0.009*** | -0.007*** | -0.008*** | -0.017*** | -0.004*** |
|  | (0.001) | (0.001) | (0.001) | (0.001) | (0.000) | (0.000) | (0.000) | (0.000) | (0.001) | (0.000) |
| high severity*z^2^ | -0.028 | -0.008 | 0.515*** | 0.095** | 0.123*** | 0.167*** | 0.216*** | 0.416*** | 0.219*** | 0.055** |
|  | (0.052) | (0.038) | (0.043) | (0.036) | (0.029) | (0.024) | (0.035) | (0.025) | (0.041) | (0.018) |
| serious*z^2^ | -0.400*** | 0.146*** | 0.346*** | 0.610*** | 0.148*** | 0.345*** | 0.382*** | 0.734*** | 0.739*** | 0.340*** |
|  | (0.050) | (0.033) | (0.051) | (0.032) | (0.021) | (0.020) | (0.057) | (0.015) | (0.028) | (0.015) |
| emergency*z^2^ | 0.041 | -0.330*** | 1.772*** | -0.007 | 0.173*** | 0.411*** | -0.980*** | 0.303*** | 0.348*** | 0.201*** |
|  | (0.056) | (0.036) | (0.052) | (0.035) | (0.025) | (0.022) | (0.045) | (0.016) | (0.029) | (0.017) |
| CCI*z^2^ | 0.101*** | 0.143*** | 0.237*** | -0.075*** | 0.036*** | 0.184*** | 0.138*** | 0.018** | 0.026 | -0.018*** |
|  | (0.008) | (0.005) | (0.022) | (0.010) | (0.006) | (0.007) | (0.027) | (0.007) | (0.017) | (0.005) |
| female*z^3^ | -0.068 | 0.006 | 0.108*** | 0.339*** | 0.039 | 0.068* | 0.163*** | 1.030*** | 0.751*** | 0.006 |
|  | (0.068) | (0.042) | (0.028) | (0.038) | (0.027) | (0.030) | (0.037) | (0.019) | (0.024) | (0.018) |
| age*z^3^ | -0.000 | 0.009*** | 0.033*** | 0.008*** | 0.007*** | 0.014*** | 0.011*** | -0.002*** | -0.002*** | 0.007*** |
|  | (0.001) | (0.001) | (0.000) | (0.001) | (0.000) | (0.000) | (0.001) | (0.000) | (0.000) | (0.000) |
| high severity*z^3^ | 0.129 | 0.654*** | 1.101*** | 0.470*** | 0.540*** | 0.413*** | 0.297*** | 0.423*** | 0.360*** | 0.498*** |
|  | (0.073) | (0.054) | (0.059) | (0.047) | (0.035) | (0.037) | (0.046) | (0.038) | (0.039) | (0.021) |
| serious*z^3^ | -0.077 | 0.669*** | 0.528*** | 0.157** | 0.385*** | 0.136*** | -0.793*** | 0.711*** | 0.411*** | 0.333*** |
|  | (0.074) | (0.064) | (0.060) | (0.049) | (0.031) | (0.036) | (0.081) | (0.029) | (0.040) | (0.020) |
| emergency*z^3^ | 1.314*** | 1.433*** | -0.298*** | 0.968*** | 0.610*** | 0.665*** | 2.106*** | 2.385*** | 1.372*** | 0.757*** |
|  | (0.102) | (0.092) | (0.056) | (0.073) | (0.053) | (0.053) | (0.096) | (0.056) | (0.059) | (0.035) |
| CCI*z^3^ | 0.041*** | -0.068*** | -0.052 | 0.249*** | 0.135*** | 0.154*** | 0.207*** | 0.308*** | 0.301*** | 0.142*** |
|  | (0.012) | (0.009) | (0.030) | (0.016) | (0.010) | (0.013) | (0.036) | (0.014) | (0.020) | (0.007) |
| *N* | 2,903,240 | 9,696,226 | 1,924,557 | 8,779,902 | 19,692,182 | 19,877,882 | 6,964,724 | 46,562,945 | 14,826,141 | 47,544,789 |

**Continued Appendix Table 1 Results of choice model**

| Variables | K29 | K80 | M47 | M50-M51 | N13 | N18 | S06 | S72 | S82 |
| --- | --- | --- | --- | --- | --- | --- | --- | --- | --- |
| dd_goodsub_z^1^*z^1^ | -0.002 | -0.058*** | -0.024*** | -0.019*** | -0.042*** | -0.066*** | -0.010*** | -0.005 | -0.006 |
|  | (0.001) | (0.002) | (0.002) | (0.002) | (0.003) | (0.003) | (0.003) | (0.004) | (0.003) |
| dd_goodsub_z^1^*(1-z^1^) | -0.007*** | -0.000 | -0.009*** | -0.011*** | -0.006 | -0.025*** | 0.023*** | 0.005 | 0.045*** |
|  | (0.002) | (0.003) | (0.001) | (0.001) | (0.004) | (0.002) | (0.004) | (0.005) | (0.005) |
| dd_poorsub_z^1^*z^1^ | 0.007** | -0.005 | -0.011*** | -0.006*** | -0.009* | -0.016*** | 0.048*** | -0.000 | 0.037*** |
|  | (0.002) | (0.003) | (0.003) | (0.002) | (0.005) | (0.003) | (0.004) | (0.006) | (0.006) |
| dd_poorsub_z^1^*(1-z^1^) | 0.008*** | -0.047*** | -0.020*** | 0.002 | -0.023*** | -0.026*** | 0.031*** | -0.001 | -0.003 |
|  | (0.002) | (0.003) | (0.003) | (0.002) | (0.004) | (0.003) | (0.004) | (0.006) | (0.005) |
| dd_goodsub_z^2^*z^2^ | -0.041*** | -0.052*** | -0.019*** | -0.019*** | -0.060*** | -0.040*** | -0.080*** | -0.057*** | -0.048*** |
|  | (0.001) | (0.001) | (0.001) | (0.001) | (0.001) | (0.002) | (0.001) | (0.002) | (0.002) |
| dd_goodsub_z^2^*(1-z^2^) | -0.010** | -0.008 | -0.034*** | -0.028*** | 0.005 | 0.038*** | -0.011** | -0.011* | -0.021*** |
|  | (0.003) | (0.004) | (0.003) | (0.002) | (0.005) | (0.004) | (0.004) | (0.005) | (0.005) |
| dd_poorsub_z^2^*z^2^ | 0.006 | 0.017*** | -0.013*** | -0.014*** | 0.018*** | 0.068*** | 0.022*** | 0.013* | -0.004 |
|  | (0.003) | (0.004) | (0.004) | (0.003) | (0.005) | (0.004) | (0.004) | (0.005) | (0.005) |
| dd_poorsub_z^2^*(1-z^2^) | -0.060*** | -0.067*** | -0.039*** | -0.043*** | -0.075*** | -0.049*** | -0.083*** | -0.076*** | -0.062*** |
|  | (0.001) | (0.001) | (0.002) | (0.001) | (0.002) | (0.002) | (0.002) | (0.002) | (0.002) |
| dd_goodsub_z^3^*z^3^ | -0.000 | 0.006** | 0.034*** | 0.023*** | -0.001 | 0.026*** | -0.027*** | 0.008* | -0.008** |
|  | (0.002) | (0.002) | (0.003) | (0.002) | (0.003) | (0.002) | (0.002) | (0.004) | (0.003) |
| dd_goodsub_z^3^*(1-z^3^) | 0.007*** | 0.043*** | -0.001 | -0.000 | 0.051*** | 0.007 | 0.026*** | -0.001 | -0.018*** |
|  | (0.002) | (0.004) | (0.002) | (0.002) | (0.006) | (0.004) | (0.005) | (0.004) | (0.004) |
| dd_poorsub_z^3^*z^3^ | -0.004 | 0.069*** | -0.004 | 0.001 | 0.079*** | 0.002 | 0.020*** | 0.011* | 0.003 |
|  | (0.003) | (0.005) | (0.003) | (0.002) | (0.006) | (0.005) | (0.005) | (0.005) | (0.005) |
| dd_poorsub_z^3^*(1-z^3^) | 0.010*** | 0.017*** | 0.032*** | 0.026*** | 0.001 | 0.037*** | -0.029*** | 0.039*** | 0.018*** |
|  | (0.002) | (0.004) | (0.003) | (0.002) | (0.004) | (0.004) | (0.003) | (0.005) | (0.004) |
| ref=male*z^1^ |  |  |  |  |  |  |  |  |  |
| female*z^1^ | 0.033 | 0.199*** | 0.213*** | 0.044* | 0.138*** | 0.077 | 0.023 | 0.023 | -0.002 |
|  | (0.023) | (0.029) | (0.023) | (0.017) | (0.037) | (0.053) | (0.052) | (0.054) | (0.045) |
| age*z^1^ | -0.001 | 0.014*** | 0.005*** | 0.009*** | 0.011*** | 0.018*** | 0.004*** | 0.003*** | 0.004*** |
|  | (0.000) | (0.001) | (0.000) | (0.000) | (0.001) | (0.001) | (0.001) | (0.001) | (0.001) |
| high severity*z^1^ | 0.296*** | -0.315*** | -0.378*** | -0.405*** | -0.379*** | -0.221** | -0.198*** | 0.083 | -0.305*** |
|  | (0.028) | (0.036) | (0.028) | (0.021) | (0.043) | (0.084) | (0.056) | (0.061) | (0.054) |
| serious*z^1^ | 0.529*** | 0.251*** | -0.265*** | 0.288*** | 0.587*** | 0.521*** | 0.737*** | 0.168** | 0.151** |
|  | (0.037) | (0.039) | (0.045) | (0.035) | (0.045) | (0.071) | (0.056) | (0.065) | (0.058) |
| emergency*z^1^ | 1.067*** | 0.333*** | 1.003*** | 0.744*** | 0.139*** | 0.958*** | 0.400*** | 0.149* | 0.238*** |
|  | (0.045) | (0.036) | (0.054) | (0.035) | (0.041) | (0.121) | (0.052) | (0.058) | (0.050) |
| CCI*z^1^ | 0.061*** | 0.036** | 0.092*** | 0.047*** | 0.145*** | -0.013 | -0.002 | 0.043 | 0.190*** |
|  | (0.012) | (0.013) | (0.015) | (0.012) | (0.028) | (0.008) | (0.032) | (0.028) | (0.042) |
| female*z^2^ | 0.121*** | -0.041 | -0.033 | -0.051** | 0.015 | -0.160*** | -0.080* | 0.094* | 0.053 |
|  | (0.023) | (0.022) | (0.025) | (0.019) | (0.031) | (0.039) | (0.038) | (0.042) | (0.037) |
| age*z^2^ | -0.006*** | 0.001** | -0.013*** | -0.009*** | 0.003*** | -0.009*** | -0.014*** | -0.011*** | -0.011*** |
|  | (0.000) | (0.000) | (0.000) | (0.000) | (0.001) | (0.001) | (0.001) | (0.001) | (0.001) |
| high severity*z^2^ | 0.215*** | 0.115*** | 0.333*** | 0.311*** | 0.122*** | 0.252*** | 0.257*** | 0.370*** | 0.430*** |
|  | (0.028) | (0.027) | (0.031) | (0.022) | (0.037) | (0.065) | (0.041) | (0.047) | (0.044) |
| serious*z^2^ | 0.232*** | 0.276*** | 0.558*** | 0.483*** | -0.135*** | 0.627*** | 0.666*** | 0.394*** | 0.546*** |
|  | (0.031) | (0.028) | (0.045) | (0.033) | (0.036) | (0.050) | (0.040) | (0.047) | (0.044) |
| emergency*z^2^ | 0.191*** | 0.116*** | 0.289*** | 0.241*** | -0.200*** | 0.804*** | 0.017 | 0.342*** | 0.315*** |
|  | (0.032) | (0.026) | (0.046) | (0.030) | (0.034) | (0.067) | (0.038) | (0.043) | (0.039) |
| CCI*z^2^ | -0.071*** | -0.009 | -0.003 | 0.028* | 0.124*** | -0.038*** | 0.019 | 0.005 | -0.005 |
|  | (0.011) | (0.010) | (0.016) | (0.013) | (0.021) | (0.006) | (0.023) | (0.020) | (0.032) |
| female*z^3^ | 0.361*** | 0.384*** | 0.148*** | 0.114*** | 0.018 | 0.294*** | 0.061 | 0.034 | -0.011 |
|  | (0.024) | (0.038) | (0.021) | (0.017) | (0.040) | (0.052) | (0.053) | (0.059) | (0.048) |
| age*z^3^ | 0.012*** | 0.015*** | 0.003*** | 0.010*** | -0.003*** | 0.016*** | 0.017*** | 0.015*** | 0.014*** |
|  | (0.000) | (0.001) | (0.000) | (0.000) | (0.001) | (0.001) | (0.001) | (0.001) | (0.001) |
| high severity*z^3^ | 0.344*** | -0.077 | 0.533*** | 0.487*** | 0.430*** | 0.697*** | 0.527*** | 0.139* | 0.477*** |
|  | (0.030) | (0.050) | (0.027) | (0.022) | (0.047) | (0.081) | (0.057) | (0.068) | (0.060) |
| serious*z^3^ | 0.141*** | 0.204*** | 0.036 | -0.532*** | -0.165** | -0.435*** | -0.088 | 0.203** | -0.054 |
|  | (0.042) | (0.061) | (0.041) | (0.035) | (0.051) | (0.068) | (0.056) | (0.076) | (0.064) |
| emergency*z^3^ | 1.145*** | 1.412*** | 0.361*** | 1.022*** | 1.408*** | 0.737*** | 0.450*** | 1.282*** | 1.308*** |
|  | (0.064) | (0.072) | (0.061) | (0.048) | (0.054) | (0.105) | (0.053) | (0.074) | (0.061) |
| CCI*z^3^ | 0.108*** | 0.124*** | 0.034* | 0.049*** | 0.071* | 0.026** | -0.104** | 0.034 | -0.109* |
|  | (0.014) | (0.020) | (0.016) | (0.014) | (0.033) | (0.008) | (0.033) | (0.032) | (0.045) |
| *N* | 12,065,316 | 13,840,747 | 18,091,406 | 29,390,872 | 6,817,923 | 2,131,450 | 2,899,154 | 2,802,555 | 3,662,722 |

Notes: (1) Z*^1^*, *Z^2^*, *Z^3^* represents whether hospital j is a public hospital, whether hospital j is a tertiary hospital, whether hospital j is a big hospital (defined as the actual number of hospital beds over the median bed for a specific disease), respectively; *female_i_* and *age_i_* denote the gender and age of inpatient *i*, respectively. *highseverity* is binary indicators of whether inpatient *i* has more than three diagnosis codes in their secondary diagnoses (then low severity as the reference group). *Serious* is binary indicators of the inpatient *i*’s urgency at admission is critical urgent or urgent (general as reference group). *emergency* indicates whether inpatient *i* is admitted through the emergency department (admission not from emergency department as reference group). *CCI* denotes Charlson Comorbidity Index (CCI) to reflect the complications of inpatients. (2) dd_goodsub_z^k^*z^k^ denotes the distance from inpatient *i*’s residence to hospital *j* minus the distance from *i*’s residence to the nearest hospital with same characteristics *Z^1^*, *Z^2^*, *Z^3^*; dd_poorsub_z^k^*z^k^ denotes the distance from inpatient *i*’s residence to hospital *j* minus the distance from *i*’s residence to the nearest hospital with different characteristics *Z^1^*, *Z^2^*, *Z^3^*.

**Appendix Table 2 Number and percentage of all kinds of hospitals for each disease**

| **Diseases**  **Variables** | **C34** | **E11** | **F20** | **I10** | **I25** | **I63** | **I84** | **J12-18** | **J20** |
| --- | --- | --- | --- | --- | --- | --- | --- | --- | --- |
| ***Hospital level*** |  |  |  |  |  |  |  |  |  |
| Primary | 53  (7.50) | 151  (12.71) | 13  (5.70) | 186  (13.61) | 229  (15.28) | 201  (14.37) | 177  (15.03) | 192  (14.24) | 199  (14.26) |
| Secondary | 305  (43.14) | 412  (34.68) | 74  (32.46) | 434  (31.75) | 423  (28.22) | 434  (31.02) | 384  (32.60) | 427  (31.68) | 417  (29.87) |
| Tertiary | 177  (25.04) | 187  (15.74) | 61  (26.75) | 187  (13.68) | 187  (12.47) | 187  (13.37) | 179  (15.20) | 186  (13.80) | 181  (12.97) |
| Un-graded | 172  (24.33) | 438  (36.87) | 80  (35.09) | 560  (40.97) | 660  (44.03) | 577  (41.24) | 438  (37.18) | 543  (40.28) | 599  (42.91) |
| ***Hospital ownership*** |  |  |  |  |  |  |  |  |  |
| Public | 391  (55.30) | 490  (41.25) | 137  (60.09) | 522  (38.19) | 496  (33.09) | 505  (36.10) | 449  (38.12) | 533  (39.54) | 511  (36.60) |
| Private | 316  (44.70) | 698  (58.75) | 91  (39.91) | 845  (61.81) | 1003  (66.91) | 894  (63.90) | 729  (61.88) | 815  (60.46) | 885  (63.40) |
| ***Whether general*** |  |  |  |  |  |  |  |  |  |
| Yes | 523  (73.97) | 864  (72.73) | 116  (50.88) | 1,023  (74.84) | 1,126  (75.12) | 1,037  (74.12) | 897  (76.15) | 1,005  (74.55) | 1,042  (74.64) |
| No | 184  (26.03) | 324  (27.27) | 112  (49.12) | 344  (25.16) | 373  (24.88) | 362  (25.88) | 281  (23.85) | 343  (25.45) | 354  (25.36) |
| ***Whether for-profit*** |  |  |  |  |  |  |  |  |  |
| Yes | 201  (28.43) | 457  (38.47) | 52  (22.81) | 540  (39.50) | 654  (43.63) | 574  (41.03) | 492  (41.77) | 532  (39.47) | 578  (41.40) |
| No | 506  (71.57) | 731  (61.53) | 176  (77.19) | 827  (60.50) | 845  (56.37) | 825  (58.97) | 686  (58.23) | 816  (60.53) | 818  (58.60) |
| ***Total number of hospitals*** | 707  (3.23) | 1,188  (5.42) | 228  (1.04) | 1,367  (6.24) | 1,499  (6.85) | 1,399  (6.39) | 1,178  (5.38) | 1,348  (6.16) | 1,396  (6.37) |

**Continued Appendix Table 2 Number and percentage of all kinds of hospitals for each disease**

| **Diseases**  **Variables** | **J44** | **K29** | **K80** | **M47** | **M50-M51** | **N13** | **N18** | **S06** | **S72** | **S82** |
| --- | --- | --- | --- | --- | --- | --- | --- | --- | --- | --- |
| ***Hospital level*** |  |  |  |  |  |  |  |  |  |  |
| Primary | 256  (15.86) | 229  (15.14) | 181  (14.43) | 232  (15.80) | 257  (16.28) | 144  (13.87) | 20  (3.96) | 49  (6.84) | 100  (10.73) | 111  (11.44) |
| Secondary | 452  (28.00) | 438  (28.95) | 418  (33.33) | 415  (28.27) | 456  (28.88) | 365  (35.16) | 226  (44.75) | 339  (47.35) | 378  (40.56) | 387  (39.90) |
| Tertiary | 185  (11.46) | 185  (12.23) | 185  (14.75) | 182  (12.40) | 185  (11.72) | 179  (17.24) | 176  (34.85) | 182  (25.42) | 181  (19.42) | 182  (18.76) |
| Un-graded | 721  (44.67) | 661  (43.69) | 470  (37.48) | 639  (43.53) | 681  (43.13) | 350  (33.72) | 83  (16.44) | 146  (20.39) | 273  (29.29) | 290  (29.90) |
| ***Hospital ownership*** |  |  |  |  |  |  |  |  |  |  |
| Public | 533  (33.02) | 530  (35.03) | 492  (39.23) | 486  (33.11) | 521  (33.00) | 440  (42.39) | 337  (66.73) | 418  (58.38) | 427  (45.82) | 447  (46.08) |
| Private | 1,081  (66.98) | 983  (64.97) | 762  (60.77) | 982  (66.89) | 1,058  (67.00) | 598  (57.61) | 168  (33.27) | 298  (41.62) | 505  (54.18) | 523  (53.92) |
| ***Whether general*** |  |  |  |  |  |  |  |  |  |  |
| Yes | 1,204  (74.60) | 1,132  (74.82) | 949  (75.68) | 1,047  (71.32) | 1,139  (72.13) | 786  (75.72) | 360  (71.29) | 504  (70.39) | 643  (68.99) | 679  (70.00) |
| No | 410  (25.40) | 381  (25.18) | 305  (24.32) | 421  (28.68) | 440  (27.87) | 252  (24.28) | 145  (28.71) | 212  (29.61) | 289  (31.01) | 291  (30.00) |
| ***Whether for-profit*** |  |  |  |  |  |  |  |  |  |  |
| Yes | 709  (43.93) | 645  (42.63) | 500  (39.87) | 644  (43.87) | 699  (44.27) | 405  (39.02) | 115  (22.77) | 195  (27.23) | 336  (36.05) | 353  (36.39) |
| No | 905  (56.07) | 868  (57.37) | 754  (60.13) | 824  (56.13) | 880  (55.73) | 633  (60.98) | 390  (77.23) | 521  (72.77) | 596  (63.95) | 617  (63.61) |
| ***Total number of hospitals*** | 1,614  (7.37) | 1,513  (6.91) | 1,254  (5.73) | 1,468  (6.70) | 1,579  (7.21) | 1,038  (4.74) | 505  (2.31) | 716  (3.27) | 932  (4.26) | 970  (4.43) |

Note: (1) Statistics shown are the number and percentage (in parentheses) of all kinds of hospitals.

**Appendix Table 3 Regression results of highly complex condition (****DWC) group**

| Variables | Highly complex condition (DWC) group | | |
| --- | --- | --- | --- |
|  | C34 | E11 | N18 |
| **HHI** | -0.612*** | 0.028 | -0.221** |
|  | (0.108) | (0.038) | (0.080) |
| **Gender** |  |  |  |
| Male (Reference) |  |  |  |
| Female | 0.001 | -0.001 | -0.072*** |
|  | (0.013) | (0.006) | (0.013) |
| Missing | -0.033 | -0.400*** | -0.038 |
|  | (0.227) | (0.030) | (0.327) |
| **Age** | -0.002** | 0.006*** | -0.001 |
|  | (0.001) | (0.000) | (0.000) |
| **Health insurance** |  |  |  |
| UEBMI (Reference) |  |  |  |
| URBMI | -0.134*** | -0.066*** | -0.247*** |
|  | (0.017) | (0.007) | (0.016) |
| NCMS | -0.170*** | -0.033*** | -0.105*** |
|  | (0.021) | (0.010) | (0.024) |
| Full self-expense | -0.288*** | -0.163*** | -0.159*** |
|  | (0.032) | (0.016) | (0.029) |
| Others | -0.021 | 0.033** | -0.080*** |
|  | (0.022) | (0.012) | (0.024) |
| **Admission source** |  |  |  |
| Others (Reference) |  |  |  |
| Emergency admission | -0.034* | 0.033*** | 0.213*** |
|  | (0.017) | (0.009) | (0.020) |
| **Urgency of admission** |  |  |  |
| General (Reference) |  |  |  |
| Critical urgent or urgent | -0.026 | 0.134*** | 0.120*** |
|  | (0.014) | (0.007) | (0.015) |
| **CCI** | -0.015*** | 0.048*** | 0.054*** |
|  | (0.002) | (0.001) | (0.002) |
| **Hospital level** |  |  |  |
| Primary (Reference) |  |  |  |
| Secondary | 0.239** | 0.119*** | -0.307*** |
|  | (0.079) | (0.026) | (0.078) |
| Tertiary | 0.401*** | 0.141*** | 0.184* |
|  | (0.084) | (0.028) | (0.082) |
| Un-grade | 0.231** | 0.010 | -0.369*** |
|  | (0.079) | (0.027) | (0.080) |
| **Hospital ownership** |  |  |  |
| Private (reference) |  |  |  |
| Public | -0.187*** | -0.086*** | -0.823*** |
|  | (0.042) | (0.015) | (0.036) |
| **Whether general** |  |  |  |
| No (Reference) |  |  |  |
| Yes | -0.020 | -0.122*** | -0.174*** |
|  | (0.016) | (0.007) | (0.017) |
| **Whether for-profit** |  |  |  |
| No (Reference) |  |  |  |
| Yes | 0.036 | -0.105*** | -0.196*** |
|  | (0.043) | (0.015) | (0.039) |
| log (number of bed) | 0.261*** | 0.193*** | 0.127*** |
|  | (0.013) | (0.005) | (0.013) |
| log (number of health personnel per 1,000 population) | 0.333*** | 0.283*** | 0.134*** |
|  | (0.022) | (0.009) | (0.020) |
| log (number of populations) | 0.047** | 0.119*** | -0.228*** |
|  | (0.015) | (0.006) | (0.016) |
| log (GDP per capita) | 0.161*** | -0.057*** | -0.079*** |
|  | (0.022) | (0.008) | (0.020) |
| *N* | 20,510 | 42,770 | 24,006 |
| *R^2^* | 0.254 | 0.313 | 0.131 |

Notes: (1) UEBM: Urban Employment Basic Medical Insurance; URBM: Urban Residents Basic Medical Insurance; NCMS: New Cooperative Medical Scheme. (2) Robust standard errors are in parentheses. (3) * *p* < 0.05, ** *p* < 0.01, *** *p* < 0.001.

**Appendix Table 4 Regression results of urgent condition** **(DWU) group**

| Variables | Urgent condition (DWU) group | | | | | |
| --- | --- | --- | --- | --- | --- | --- |
|  | I63 | K80 | N13 | S06 | S72 | S82 |
| **HHI** | 0.044 | -0.010 | 0.185* | 0.113 | -0.063 | -0.161 |
|  | (0.036) | (0.038) | (0.076) | (0.059) | (0.139) | (0.127) |
| **Gender** |  |  |  |  |  |  |
| Male (Reference) |  |  |  |  |  |  |
| Female | -0.046*** | 0.038*** | 0.056*** | -0.174*** | 0.039* | -0.095*** |
|  | (0.005) | (0.006) | (0.009) | (0.014) | (0.018) | (0.015) |
| Missing | 0.120 | -0.605 | 0.000 | -0.166 | -0.029 | 1.146*** |
|  | (0.278) | (1.037) | (.) | (0.316) | (0.038) | (0.266) |
| **Age** | 0.001*** | 0.002*** | 0.009*** | 0.011*** | -0.000 | 0.006*** |
|  | (0.000) | (0.000) | (0.000) | (0.000) | (0.001) | (0.000) |
| **Health insurance** |  |  |  |  |  |  |
| UEBMI (Reference) |  |  |  |  |  |  |
| URBMI | -0.087*** | -0.030*** | -0.031** | -0.007 | -0.087*** | -0.022 |
|  | (0.006) | (0.008) | (0.011) | (0.031) | (0.025) | (0.023) |
| NCMS | -0.051*** | -0.008 | -0.004 | 0.056 | -0.084** | 0.001 |
|  | (0.008) | (0.009) | (0.014) | (0.033) | (0.031) | (0.027) |
| Full self-expense | -0.205*** | -0.151*** | -0.351*** | -0.013 | -0.214*** | 0.027 |
|  | (0.014) | (0.014) | (0.019) | (0.030) | (0.033) | (0.025) |
| Others | 0.021* | -0.114*** | 0.042* | 0.170*** | -0.090** | 0.130*** |
|  | (0.010) | (0.012) | (0.019) | (0.033) | (0.034) | (0.028) |
| **Admission source** |  |  |  |  |  |  |
| Others (Reference) |  |  |  |  |  |  |
| Emergency admission | 0.051*** | -0.076*** | -0.176*** | -0.048** | 0.037* | 0.100*** |
|  | (0.006) | (0.007) | (0.011) | (0.015) | (0.019) | (0.017) |
| **Urgency of admission** |  |  |  |  |  |  |
| General (Reference) |  |  |  |  |  |  |
| Critical urgent or urgent | 0.128*** | -0.026** | -0.175*** | 0.427*** | 0.083*** | 0.123*** |
|  | (0.005) | (0.008) | (0.011) | (0.015) | (0.020) | (0.018) |
| **CCI** | 0.080*** | 0.073*** | 0.122*** | 0.166*** | 0.054*** | 0.099*** |
|  | (0.002) | (0.002) | (0.007) | (0.009) | (0.008) | (0.011) |
| **Hospital level** |  |  |  |  |  |  |
| Primary (Reference) |  |  |  |  |  |  |
| Secondary | 0.071*** | 0.327*** | -0.013 | -0.067 | 0.128* | 0.186*** |
|  | (0.015) | (0.024) | (0.019) | (0.072) | (0.063) | (0.043) |
| Tertiary | 0.102*** | 0.426*** | 0.105*** | -0.182* | 0.187** | 0.251*** |
|  | (0.018) | (0.026) | (0.025) | (0.078) | (0.071) | (0.053) |
| Un-grade | 0.056*** | 0.175*** | 0.063*** | 0.093 | 0.009 | 0.049 |
|  | (0.015) | (0.024) | (0.016) | (0.076) | (0.064) | (0.040) |
| **Hospital ownership** |  |  |  |  |  |  |
| Private (reference) |  |  |  |  |  |  |
| Public | 0.005 | -0.056*** | -0.028 | 0.022 | -0.008 | -0.096** |
|  | (0.010) | (0.016) | (0.017) | (0.040) | (0.040) | (0.031) |
| **Whether general** |  |  |  |  |  |  |
| No (Reference) |  |  |  |  |  |  |
| Yes | -0.101*** | -0.061*** | 0.021 | -0.041* | -0.120*** | -0.062*** |
|  | (0.006) | (0.007) | (0.011) | (0.019) | (0.020) | (0.016) |
| **Whether for-profit** |  |  |  |  |  |  |
| No (Reference) |  |  |  |  |  |  |
| Yes | -0.068*** | 0.034* | 0.120*** | 0.099* | 0.114** | 0.095*** |
|  | (0.010) | (0.016) | (0.014) | (0.041) | (0.041) | (0.029) |
| log (number of bed) | 0.276*** | 0.219*** | 0.342*** | 0.495*** | 0.309*** | 0.308*** |
|  | (0.005) | (0.006) | (0.008) | (0.015) | (0.018) | (0.015) |
| log (number of health personnel per 1,000 population) | 0.183*** | 0.203*** | 0.340*** | 0.227*** | 0.253*** | 0.258*** |
|  | (0.008) | (0.009) | (0.014) | (0.025) | (0.030) | (0.025) |
| log (number of populations) | 0.118*** | 0.071*** | 0.084*** | 0.025 | 0.034 | 0.103*** |
|  | (0.006) | (0.006) | (0.009) | (0.015) | (0.019) | (0.015) |
| log (GDP per capita) | 0.065*** | -0.018* | -0.023 | -0.014 | -0.058* | -0.107*** |
|  | (0.008) | (0.009) | (0.012) | (0.022) | (0.028) | (0.023) |
| *N* | 79,413 | 59,139 | 36,344 | 23,628 | 17,247 | 22,051 |
| *R^2^* | 0.334 | 0.204 | 0.318 | 0.229 | 0.119 | 0.158 |

Notes: (1) UEBM: Urban Employment Basic Medical Insurance; URBM: Urban Residents Basic Medical Insurance; NCMS: New Cooperative Medical Scheme. (2) Robust standard errors are in parentheses. (3) * *p* < 0.05, ** *p* < 0.01, *** *p* < 0.001.

**Appendix Table 5 Regression results of less complex and less urgent condition** **(DWL) group**

| Variables | Less complex and less urgent condition (DWL) group | | | | | | | | | |
| --- | --- | --- | --- | --- | --- | --- | --- | --- | --- | --- |
|  | F20 | I10 | I25 | I84 | J12-J18 | J20 | J44 | K29 | M47 | M50-M51 |
| **HHI** | 0.836*** | 0.320*** | 0.050 | 0.533*** | 0.257*** | 0.084*** | 0.196*** | 0.594*** | 0.283*** | 0.364*** |
|  | (0.042) | (0.034) | (0.044) | (0.071) | (0.010) | (0.024) | (0.022) | (0.029) | (0.043) | (0.033) |
| **Gender** |  |  |  |  |  |  |  |  |  |  |
| Male (Reference) |  |  |  |  |  |  |  |  |  |  |
| Female | -0.112*** | -0.027*** | -0.071*** | 0.016** | -0.033*** | 0.001 | -0.030*** | 0.009 | 0.033*** | 0.019*** |
|  | (0.008) | (0.006) | (0.005) | (0.006) | (0.003) | (0.004) | (0.003) | (0.005) | (0.005) | (0.004) |
| Missing | 0.000 | -0.229* | 0.000 | 0.467*** | -0.362** | -0.286** | 0.045 | 0.601*** | 0.256*** | -0.228 |
|  | (.) | (0.102) | (.) | (0.029) | (0.130) | (0.110) | (0.126) | (0.117) | (0.058) | (0.273) |
| **Age** | 0.001 | 0.007*** | 0.003*** | -0.001*** | 0.010*** | 0.010*** | 0.003*** | 0.010*** | 0.003*** | 0.004*** |
|  | (0.000) | (0.000) | (0.000) | (0.000) | (0.000) | (0.000) | (0.000) | (0.000) | (0.000) | (0.000) |
| **Health insurance** |  |  |  |  |  |  |  |  |  |  |
| UEBMI (Reference) |  |  |  |  |  |  |  |  |  |  |
| URBMI | -0.189*** | -0.093*** | -0.061*** | -0.054*** | -0.064*** | -0.076*** | -0.102*** | -0.116*** | -0.097*** | -0.065*** |
|  | (0.013) | (0.007) | (0.006) | (0.007) | (0.005) | (0.007) | (0.004) | (0.007) | (0.005) | (0.005) |
| NCMS | -0.233*** | -0.025** | -0.043*** | -0.028** | 0.017** | -0.023** | -0.090*** | -0.086*** | -0.105*** | -0.034*** |
|  | (0.014) | (0.010) | (0.008) | (0.009) | (0.006) | (0.009) | (0.005) | (0.009) | (0.008) | (0.006) |
| Full self-expense | -0.362*** | -0.180*** | -0.101*** | -0.259*** | -0.096*** | -0.164*** | -0.188*** | -0.268*** | -0.160*** | -0.119*** |
|  | (0.029) | (0.017) | (0.015) | (0.019) | (0.006) | (0.010) | (0.010) | (0.014) | (0.020) | (0.016) |
| Others | -0.107*** | 0.057*** | 0.018 | -0.019 | -0.006 | -0.032** | -0.003 | -0.025* | 0.008 | 0.065*** |
|  | (0.019) | (0.013) | (0.010) | (0.014) | (0.007) | (0.010) | (0.006) | (0.010) | (0.011) | (0.009) |
| **Admission source** |  |  |  |  |  |  |  |  |  |  |
| Others (Reference) |  |  |  |  |  |  |  |  |  |  |
| Emergency admission | -0.078*** | -0.038*** | -0.003 | 0.004 | 0.034*** | 0.008 | 0.027*** | -0.072*** | -0.032** | 0.013 |
|  | (0.015) | (0.008) | (0.007) | (0.011) | (0.003) | (0.006) | (0.004) | (0.008) | (0.010) | (0.008) |
| **Urgency of admission** |  |  |  |  |  |  |  |  |  |  |
| General (Reference) |  |  |  |  |  |  |  |  |  |  |
| Critical urgent or urgent | -0.149*** | 0.009 | 0.073*** | -0.039** | 0.129*** | 0.073*** | 0.105*** | 0.053*** | 0.001 | -0.029*** |
|  | (0.015) | (0.007) | (0.005) | (0.012) | (0.003) | (0.005) | (0.003) | (0.007) | (0.008) | (0.007) |
| **CCI** | 0.082*** | 0.091*** | 0.066*** | 0.043*** | 0.106*** | 0.091*** | 0.079*** | 0.080*** | 0.072*** | 0.066*** |
|  | (0.006) | (0.002) | (0.001) | (0.007) | (0.002) | (0.003) | (0.001) | (0.002) | (0.003) | (0.003) |
| **Hospital level** |  |  |  |  |  |  |  |  |  |  |
| Primary (Reference) |  |  |  |  |  |  |  |  |  |  |
| Secondary | 0.033 | 0.049** | 0.005 | 0.256*** | 0.067*** | 0.141*** | 0.115*** | 0.070*** | 0.120*** | 0.066*** |
|  | (0.025) | (0.018) | (0.012) | (0.016) | (0.013) | (0.013) | (0.008) | (0.013) | (0.009) | (0.009) |
| Tertiary | -0.036 | 0.029 | 0.038** | 0.291*** | 0.023 | 0.100*** | 0.124*** | -0.002 | 0.146*** | 0.134*** |
|  | (0.029) | (0.021) | (0.014) | (0.020) | (0.014) | (0.016) | (0.009) | (0.016) | (0.013) | (0.012) |
| Un-grade | 0.062** | -0.003 | -0.077*** | 0.074*** | 0.026* | 0.031** | -0.042*** | 0.010 | 0.015* | 0.037*** |
|  | (0.021) | (0.019) | (0.011) | (0.015) | (0.013) | (0.011) | (0.007) | (0.011) | (0.007) | (0.007) |
| **Hospital ownership** |  |  |  |  |  |  |  |  |  |  |
| Private (reference) |  |  |  |  |  |  |  |  |  |  |
| Public | 0.287*** | -0.038** | -0.001 | -0.148*** | 0.127*** | 0.025* | 0.053*** | -0.045*** | -0.058*** | -0.107*** |
|  | (0.013) | (0.012) | (0.009) | (0.014) | (0.007) | (0.010) | (0.006) | (0.011) | (0.008) | (0.007) |
| **Whether general** |  |  |  |  |  |  |  |  |  |  |
| No (Reference) |  |  |  |  |  |  |  |  |  |  |
| Yes | -0.261*** | -0.109*** | -0.092*** | -0.111*** | -0.101*** | -0.040*** | -0.059*** | -0.142*** | -0.040*** | -0.080*** |
|  | (0.012) | (0.007) | (0.005) | (0.007) | (0.003) | (0.005) | (0.003) | (0.006) | (0.005) | (0.004) |
| **Whether for-profit** |  |  |  |  |  |  |  |  |  |  |
| No (Reference) |  |  |  |  |  |  |  |  |  |  |
| Yes | 0.258*** | -0.043*** | -0.039*** | -0.147*** | 0.060*** | 0.067*** | 0.038*** | 0.060*** | 0.007 | 0.013* |
|  | (0.015) | (0.012) | (0.009) | (0.012) | (0.008) | (0.009) | (0.005) | (0.010) | (0.006) | (0.006) |
| log (number of bed) | 0.300*** | 0.190*** | 0.243*** | 0.115*** | 0.184*** | 0.178*** | 0.209*** | 0.220*** | 0.213*** | 0.239*** |
|  | (0.008) | (0.005) | (0.004) | (0.006) | (0.003) | (0.004) | (0.003) | (0.005) | (0.004) | (0.004) |
| log (number of health personnel per 1,000 population) | -0.005 | 0.210*** | 0.257*** | 0.192*** | 0.356*** | 0.218*** | 0.173*** | 0.287*** | 0.254*** | 0.284*** |
|  | (0.015) | (0.009) | (0.008) | (0.010) | (0.004) | (0.006) | (0.005) | (0.009) | (0.008) | (0.007) |
| log (number of populations) | -0.033*** | 0.135*** | 0.109*** | 0.085*** | 0.094*** | 0.059*** | 0.086*** | 0.108*** | 0.070*** | 0.105*** |
|  | (0.009) | (0.007) | (0.005) | (0.008) | (0.003) | (0.004) | (0.003) | (0.005) | (0.005) | (0.004) |
| log (GDP per capita) | 0.019 | -0.004 | -0.022** | 0.101*** | 0.003 | -0.008 | 0.068*** | -0.122*** | -0.004 | 0.001 |
|  | (0.014) | (0.008) | (0.007) | (0.009) | (0.004) | (0.006) | (0.004) | (0.007) | (0.007) | (0.006) |
| *N* | 47,022 | 36,575 | 76,196 | 35,331 | 179,614 | 62,456 | 151,566 | 58,148 | 59,027 | 99,761 |
| *R^2^* | 0.177 | 0.331 | 0.335 | 0.203 | 0.490 | 0.314 | 0.372 | 0.290 | 0.316 | 0.277 |

Notes: (1) UEBM: Urban Employment Basic Medical Insurance; URBM: Urban Residents Basic Medical Insurance; NCMS: New Cooperative Medical Scheme. (2) Robust standard errors are in parentheses. (3) * *p* < 0.05, ** *p* < 0.01, *** *p* < 0.001.

**Appendix Table 6 Regression results adding interaction terms (continuous variables)**

| Variables | (1) | (2) | (3) |
| --- | --- | --- | --- |
| HHI | 0.252*** | 0.540*** | 0.303*** |
|  | (0.007) | (0.019) | (0.010) |
| Emergency rate | 4.675*** | 4.816*** | 4.673*** |
|  | (0.164) | (0.164) | (0.164) |
| Average CCI | 0.190*** | 0.194*** | 0.193*** |
|  | (0.012) | (0.012) | (0.012) |
| Emergency rate*HHI | - | -1.384*** | - |
|  | - | (0.090) | - |
| Average CCI*HHI | - | - | -0.060*** |
|  | - | - | (0.008) |
| Other control variables | Yes | Yes | Yes |
| *N* | 1,130,804 | 1,130,804 | 1,130,804 |
| *R^2^* | 0.422 | 0.422 | 0.422 |

Notes: (1) Robust standard errors are in parentheses. (2) Other control variables include gender, age, health insurance program, the urgency of admission, the hospital level, ownership, whether general, whether for-profit, the number of the bed, the number of health personnel per 1,000 population, the number of populations, the GDP per capita, and a set of dummy variables of first three digits of ICD-10 codes. (3) * *p* < 0.05, ** *p* < 0.01, *** *p* < 0.001.

**Appendix Table 7 Regression results adding interaction terms (categorical variables)**

| Variables | (1) | (2) | (3) |
| --- | --- | --- | --- |
| **HHI** | 0.252*** | 0.703*** | 0.492*** |
|  | (0.007) | (0.021) | (0.015) |
| **Emergency rate** **quantile** |  |  |  |
| 0-25% (reference) |  |  |  |
| 25%-75% | -0.578*** | -0.565*** | -0.581*** |
|  | (0.012) | (0.012) | (0.012) |
| 75%-100% | 0.759*** | 0.782*** | 0.756*** |
|  | (0.008) | (0.008) | (0.008) |
| **Average CCI quantile** |  |  |  |
| 0-25% (reference) |  |  |  |
| 25%-75% | 0.237*** | 0.239*** | 0.253*** |
|  | (0.011) | (0.011) | (0.011) |
| 75%-100% | 1.055*** | 1.058*** | 1.073*** |
|  | (0.013) | (0.013) | (0.013) |
| **Emergency rate quantile*HHI** |  |  |  |
| 0-25%*HHI (reference) |  |  |  |
| 25%-75%*HHI | - | -0.417*** | - |
|  | - | (0.022) | - |
| 75%-100%*HHI | - | -0.558*** | - |
|  | - | (0.021) | - |
| **Average CCI quantile*HHI** |  |  |  |
| 0-25%*HHI (reference) |  |  |  |
| 25%-75%*HHI | - | - | -0.289*** |
|  | - | - | (0.016) |
| 75%-100%*HHI | - | - | -0.362*** |
|  | - | - | (0.023) |
| **Other control variables** | Yes | Yes | Yes |
| *N* | 1,130,804 | 1,130,804 | 1,130,804 |
| *R^2^* | 0.422 | 0.423 | 0.422 |

Notes: (1) Robust standard errors are in parentheses. (2) Other control variables include gender, age, health insurance program, the urgency of admission, the hospital level, ownership, whether general, whether for-profit, the number of the bed, the number of health personnel per 1,000 population, the number of populations, the GDP per capita, and a set of dummy variables of first three digits of ICD-10 codes. (3) * *p* < 0.05, ** *p* < 0.01, *** *p* < 0.001.

Appendix Figures:


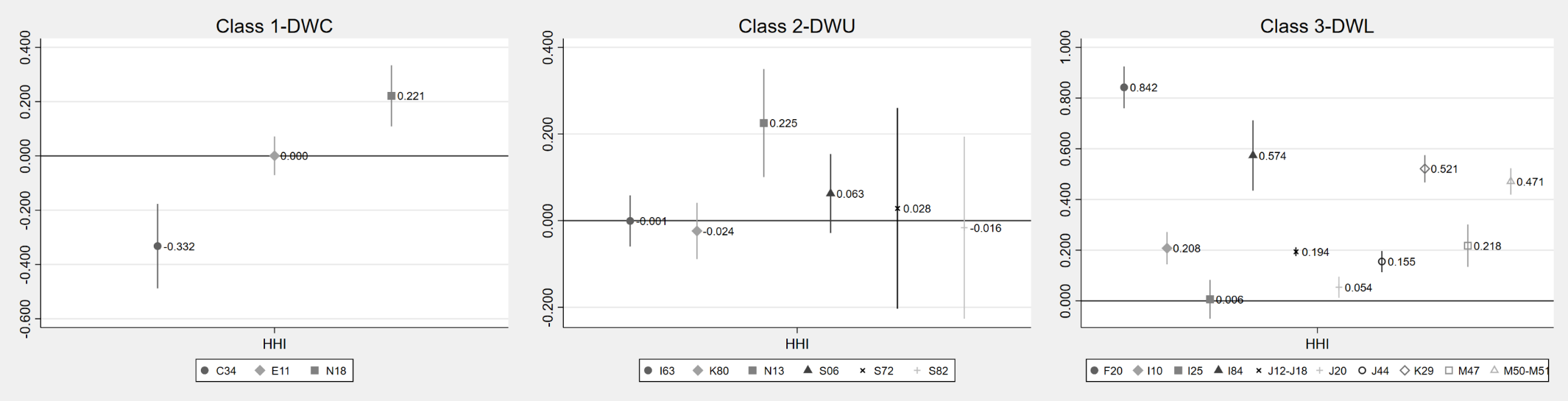


**Appendix Figure 1 Regression analysis controlling LOS and inpatient mortality**

Note: (1) DWC group refers to diseases with highly complex conditions. DWU group refers to diseases with urgent conditions. DWL group refers to diseases with less complex and urgent conditions. (2) C34: bronchial and lung cancer. E11: non-insulin-dependent diabetes. N18: chronic kidney disease. (3) I63: cerebral infarction. K80: cholelithiasis. N13: obstructive and reflux uropathy. S06: intracranial injury. S72: femoral fracture. S82: fractures of the lower leg (including the ankle). (4) F20: schizophrenia. I10: essential hypertension. I25: chronic ischemic heart disease. I84: hemorrhoids. J12-J18: pneumonia. J20: acute bronchitis. J44: chronic obstructive pulmonary disease. K29: gastritis and duodenitis. M47: spine joint stiffness. M50-M51: intervertebral disc disorders.


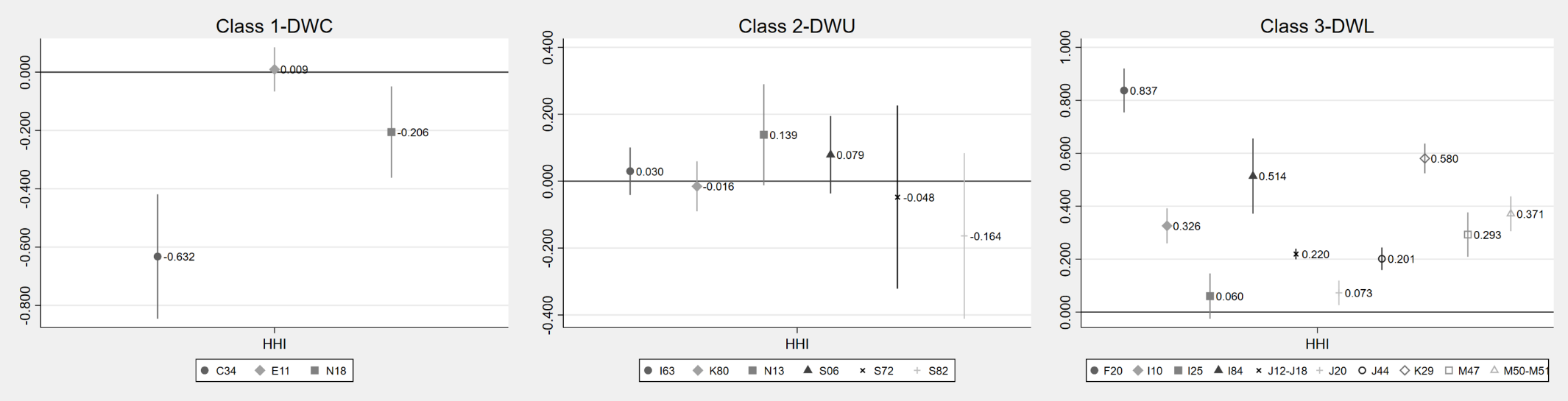


**Appendix Figure 2 Regression analysis controlling a set of dummy variables of the complete ICD-10 code of primary diagnosis**

Note: (1) DWC group refers to diseases with highly complex conditions. DWU group refers to diseases with urgent conditions. DWL group refers to diseases with less complex and urgent conditions. (2) C34: bronchial and lung cancer. E11: non-insulin-dependent diabetes. N18: chronic kidney disease. (3) I63: cerebral infarction. K80: cholelithiasis. N13: obstructive and reflux uropathy. S06: intracranial injury. S72: femoral fracture. S82: fractures of the lower leg (including the ankle). (4) F20: schizophrenia. I10: essential hypertension. I25: chronic ischemic heart disease. I84: hemorrhoids. J12-J18: pneumonia. J20: acute bronchitis. J44: chronic obstructive pulmonary disease. K29: gastritis and duodenitis. M47: spine joint stiffness. M50-M51: intervertebral disc disorders.


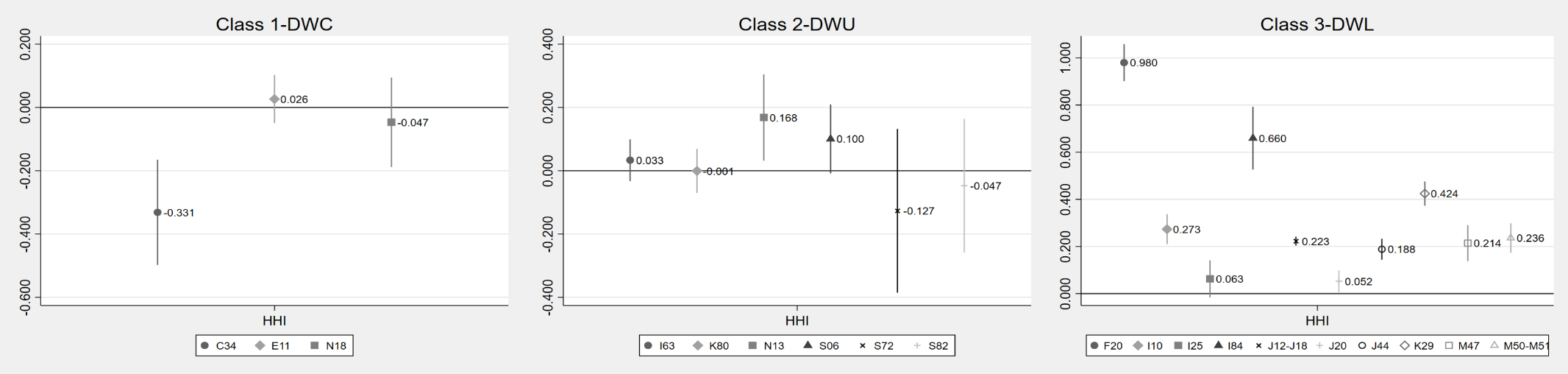


**Appendix Figure 3 Regression analysis: 80km**

Note: (1) DWC group refers to diseases with highly complex conditions. DWU group refers to diseases with urgent conditions. DWL group refers to diseases with less complex and urgent conditions. (2) C34: bronchial and lung cancer. E11: non-insulin-dependent diabetes. N18: chronic kidney disease. (3) I63: cerebral infarction. K80: cholelithiasis. N13: obstructive and reflux uropathy. S06: intracranial injury. S72: femoral fracture. S82: fractures of the lower leg (including the ankle). (4) F20: schizophrenia. I10: essential hypertension. I25: chronic ischemic heart disease. I84: hemorrhoids. J12-J18: pneumonia. J20: acute bronchitis. J44: chronic obstructive pulmonary disease. K29: gastritis and duodenitis. M47: spine joint stiffness. M50-M51: intervertebral disc disorders.


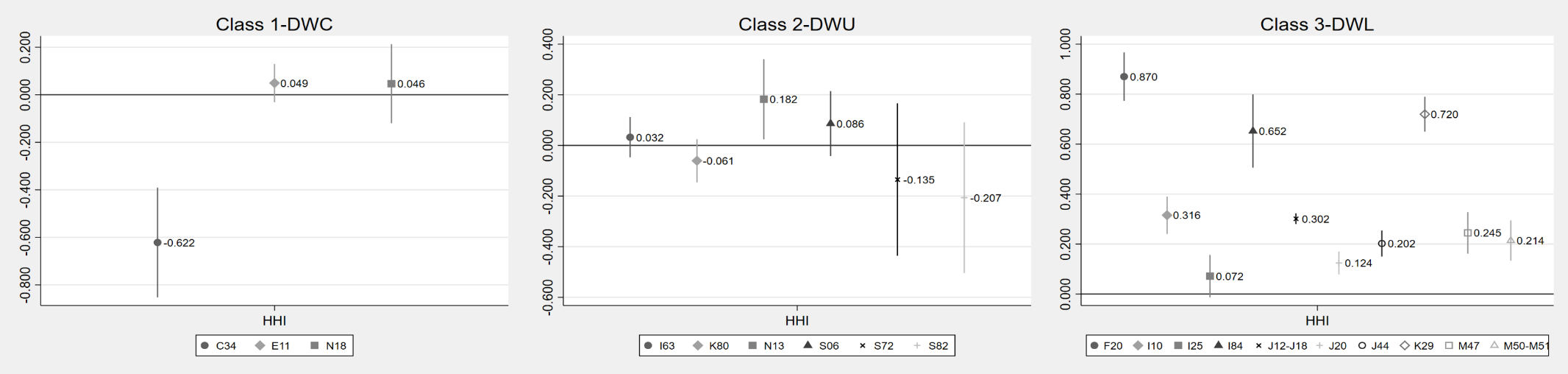


**Appendix Figure 4 Regression analysis: 150km**

Note: (1) DWC group refers to diseases with highly complex conditions. DWU group refers to diseases with urgent conditions. DWL group refers to diseases with less complex and urgent conditions. (2) C34: bronchial and lung cancer. E11: non-insulin-dependent diabetes. N18: chronic kidney disease. (3) I63: cerebral infarction. K80: cholelithiasis. N13: obstructive and reflux uropathy. S06: intracranial injury. S72: femoral fracture. S82: fractures of the lower leg (including the ankle). (4) F20: schizophrenia. I10: essential hypertension. I25: chronic ischemic heart disease. I84: hemorrhoids. J12-J18: pneumonia. J20: acute bronchitis. J44: chronic obstructive pulmonary disease. K29: gastritis and duodenitis. M47: spine joint stiffness. M50-M51: intervertebral disc disorders.


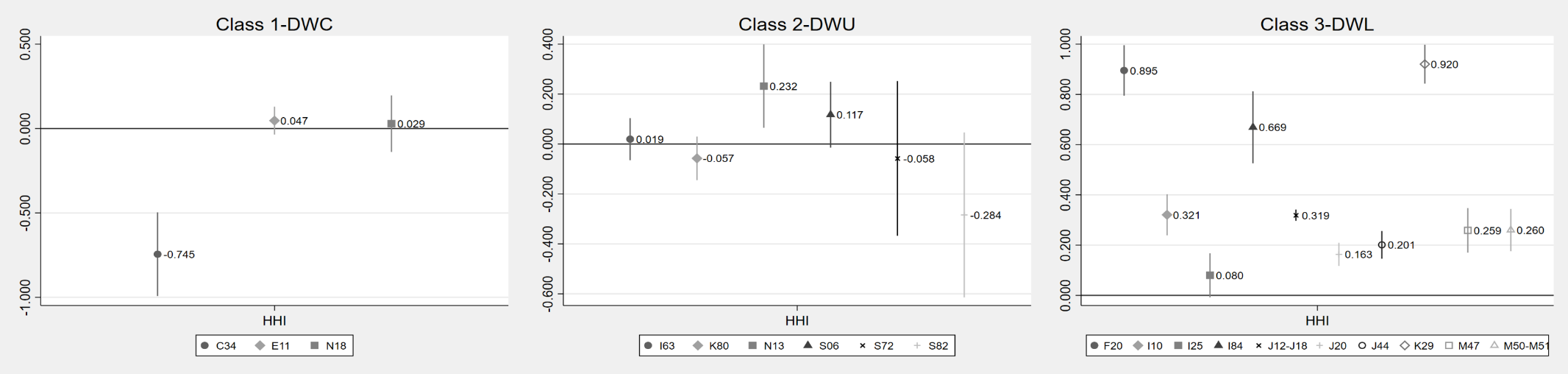


**Appendix Figure 5 Regression analysis: 200km**

Note: (1) DWC group refers to diseases with highly complex conditions. DWU group refers to diseases with urgent conditions. DWL group refers to diseases with less complex and urgent conditions. (2) C34: bronchial and lung cancer. E11: non-insulin-dependent diabetes. N18: chronic kidney disease. (3) I63: cerebral infarction. K80: cholelithiasis. N13: obstructive and reflux uropathy. S06: intracranial injury. S72: femoral fracture. S82: fractures of the lower leg (including the ankle). (4) F20: schizophrenia. I10: essential hypertension. I25: chronic ischemic heart disease. I84: hemorrhoids. J12-J18: pneumonia. J20: acute bronchitis. J44: chronic obstructive pulmonary disease. K29: gastritis and duodenitis. M47: spine joint stiffness. M50-M51: intervertebral disc disorders.

**Reference**

1. Wang X, Yang H, Duan Z, Pan J: **Spatial accessibility of primary health care in China: a case study in Sichuan Province**. *Soc Sci Med* 2018, **209**:14-24.

2. Lu L, Pan J: **Does hospital competition lead to medical equipment expansion? Evidence on the medical arms race**. *Health Care Manage Sci* 2021:1-15.

3. Lu L, Chen T, Lan T, Pan J: **The Comparison Between Different Hospital Market Definition Approaches: An Empirical Analysis of 11 Representative Diseases in Sichuan Province, China**. *Frontiers in Public Health* 2021, **9**(1165).

4. Kessler DP, Mcclellan MB: **Is hospital competition socially wasteful?** *The Quarterly Journal of Economics* 2000, **115**(2):577-615.

5. Lin X, Cai M, Fu Q, He K, Jiang T, Lu W, Ni Z, Tao H: **Does Hospital Competition Harm Inpatient Quality? Empirical Evidence from Shanxi, China**. *Int J Env Res Public Health* 2018, **15**(10):2283.

6. Gaynor M, Moreno-Serra R, Propper C: **Death by market power: reform, competition, and patient outcomes in the National Health Service**. *Am Econ J-Econ Policy* 2013, **5**(4):134-166.

7. Gowrisankaran G, Town RJ: **Competition, payers, and hospital quality**. *Health Services Res* 2003, **38**(6p1):1403-1422.

8. Colla C, Bynum J, Austin A, Skinner J: **Hospital competition, quality, and expenditures in the US Medicare population**. In*.*: National Bureau of Economic Research; 2016.

9. Grant RW, McCloskey J, Hatfield M, Uratsu C, Ralston JD, Bayliss E, Kennedy CJ: **Use of Latent Class Analysis and k-Means Clustering to Identify Complex Patient Profiles**. *JAMA Network Open* 2020, **3**(12):e2029068-e2029068.

10. Jackson T, Dimitropoulos V, Madden R, Gillett S: **Australian diagnosis related groups: Drivers of complexity adjustment**. *Health Policy* 2015, **119**(11):1433-1441.

11. Chen J, Yang L, Qian Z, Sun M, Yu H, Ma X, Wan C, Yang Y: **Cluster analysis of differences in medical economic burden among residents of different economic levels in Guangdong Province, China**. *BMC Health Services Research* 2020, **20**(1):1-11.

12. Rosner B: **Fundamentals of biostatistics**: Nelson Education; 2015.
